# Accelerated remodeling of the mesophyll-bundle sheath interface in the maize C4 cycle mutant leaves

**DOI:** 10.1038/s41598-022-09135-7

**Published:** 2022-03-23

**Authors:** Peng Gao, Pengfei Wang, Baijuan Du, Pinghua Li, Byung-Ho Kang

**Affiliations:** 1 State Key Laboratory of Agrobiotechnology, Center for Cell and Developmental Biology, School of Life Sciences, The Chinese University of Hong Kong, Hong Kong, China; 2 State Key Laboratory of Crop Biology, College of Agronomic Sciences, Shandong Agricultural University, Tai’an, 271018 China

**Keywords:** Cell biology, Plant sciences

## Abstract

C4 photosynthesis in the maize leaf involves the exchange of organic acids between mesophyll (M) and the bundle sheath (BS) cells. The transport is mediated by plasmodesmata embedded in the suberized cell wall. We examined the maize Kranz anatomy with a focus on the plasmodesmata and cell wall suberization with microscopy methods. In the young leaf zone where M and BS cells had indistinguishable proplastids, plasmodesmata were simple and no suberin was detected. In leaf zones where dimorphic chloroplasts were evident, the plasmodesma acquired sphincter and cytoplasmic sleeves, and suberin was discerned. These modifications were accompanied by a drop in symplastic dye mobility at the M-BS boundary. We compared the kinetics of chloroplast differentiation and the modifications in M-BS connectivity in *ppdk* and *dct2* mutants where C4 cycle is affected. The rate of chloroplast diversification did not alter, but plasmodesma remodeling, symplastic transport inhibition, and cell wall suberization were observed from younger leaf zone in the mutants than in wild type. Our results indicate that inactivation of the C4 genes accelerated the changes in the M-BS interface, and the reduced permeability suggests that symplastic transport between M and BS could be regulated for normal operation of C4 cycle.

## Introduction

C4 photosynthesis involves a biochemical cycle that enrich CO_2_ around Rubisco to suppress energetically wasteful photorespiration^1,2^. The cycle requires two compartments, one for producing four carbon (C4) acids through fixation of atmospheric CO_2_ and the other for decarboxylating the C4 acids to provide Rubisco with CO_2_. The Kranz anatomy is the leaf architecture that partitions the two compartments into two cell types^3,4^.

In the maize leaf, bundle sheath (BS) cells form a cylinder surrounding the vascular cells, and the mesophyll (M) cells are located outside the BS^5^. The classical NADP-malic enzyme-mediated C4 pathway occurs in maize. Phosphoenolpyruvate carboxylase in the M cell cytosol captures CO_2_ to produce oxaloacetate, which is subsequently reduced to malate in the M chloroplast. Malate moves to the BS cell and is subsequently imported into the BS chloroplast by DCT2, a dicarboxylic acid transporter. Decarboxylation of malate produces pyruvate and CO_2_ and pyruvate returns to the M cell. The pyruvate orthophosphate dikinase PPDK regenerates phosphoenolpyruvate from pyruvate in the M chloroplast for another round of CO_2_ pumping^6^. Maize mutant lines in which genes encoding DCT2 or PPDK involved in C4 cycle are inactivated have been characterized^7,8^. C4 photosynthesis is defective in the mutant plants, but their Kranz anatomy is not compromised.

The M and BS chloroplasts of the maize have different energy requirements and have different ultrastructural features^9,10^ . In the M chloroplast, both photosystem II (PSII) and photosystem I (PSI) mediate linear electron flow to produce ATP and NADPH. The BS chloroplast produces ATP via PSI-mediated cyclic electron flow since reducing power comes with malate that originates in M cells. To accommodate PSII and PSI complexes, the M chloroplasts have grana stacks and stroma lamellae. Thylakoids in BS chloroplasts consist of unstacked thylakoids because they lack PSII^11–13^. Proplastids in immature cells at the maize leaf base are morphologically similar, indicating that the dimorphic chloroplasts arise as the two cell types mature along the leaf developmental gradient toward the tip^14^.

Plasmodesmata (PD) are channels through the cell wall that connect the cytoplasms of neighboring plant cells. Transmission electron microscopy (TEM) studies have revealed that each channel consists of the plasma membrane and a membrane tubule called the desmotubule that originates from the endoplasmic reticulum^15,16^. Metabolites, signaling molecules, and small proteins can diffuse into adjacent cells through the cytoplasmic sleeve, the gap between the plasma membrane and the desmotubule^17–19^. Callose, a β-1,3-glucan, is associated with PD, and increases in callose amounts cause constriction of the PD to inhibit diffusion^20,21^. PD are assembled in the cell plate during cytokinesis, and the nascent PD are morphologically simple. As the cells that they connect differentiate, PD become mature, and their structures elaborate^15^. PD density in a plant tissue also varies in relation to the rate of symplastic flux in the tissue.

The PD at the M-BS interface in the maize leaf mediate the transport of molecular components of the C4 cycle^22,23^. The movement of malate to the BS cell and recycling of pyruvate to the M cell are dependent on passive transport^24^ . Transamination of oxaloacetate generates aspartate in the M cytosol, and this C4 acid also enters BS through PD^25,26^. The PD density in the maize leaf is approximately 9 times higher than in the rice leaf, a C3 crop plant. The high degree of symplastic connectivity of the PD in maize reflects its significance in solute exchange in the C4 system^27^. It has been shown that PD linking M and BS cells have a sphincter on the M side in dual-cell C4 plants including maize and sugarcane^28–30^.

Another unique feature at the M-BS boundary is the suberin lamella^31^. Suberin is one of lipophilic biopolymers of land plants, constituting a lamella immediate outside the plasma membrane^32^ and the suberin coating is often associated with the vascular tissue^33,34^. The BS is the outermost cell layer of the vascular bundle in the maize leaf and its cell wall is supplemented with suberin^35,36^. Because sucrose in the vascular parenchyma enters the companion cell after being exported to the apoplast, the hydrophobic stratum of the vascular bundle may inhibit the loss of sucrose^37,38^. It was recently shown that the suberin layer in the *Setaria* BS cell wall is critical for keeping CO_2_ from leaking away from BS^39^.

Despite the solute exchange through the M-BS interface being a critical component of the dual cell C4 cycle, it has not been investigated how the intercellular transport machinery is assembled and regulated in relation to the maize C4 leaf development. Using a suite of microscopy methods combined with transcriptomic analyses, we provide evidence that mutation of genes involved in the synthesis or transport of C4 metabolites, thereby inhibiting accumulation of CO_2_ in BS cells, stimulates PD development and construction of suberin barrier in younger M-BS pairs.

## Results

### Development of dimorphic chloroplasts in the maize leaf

**Figure 1:**
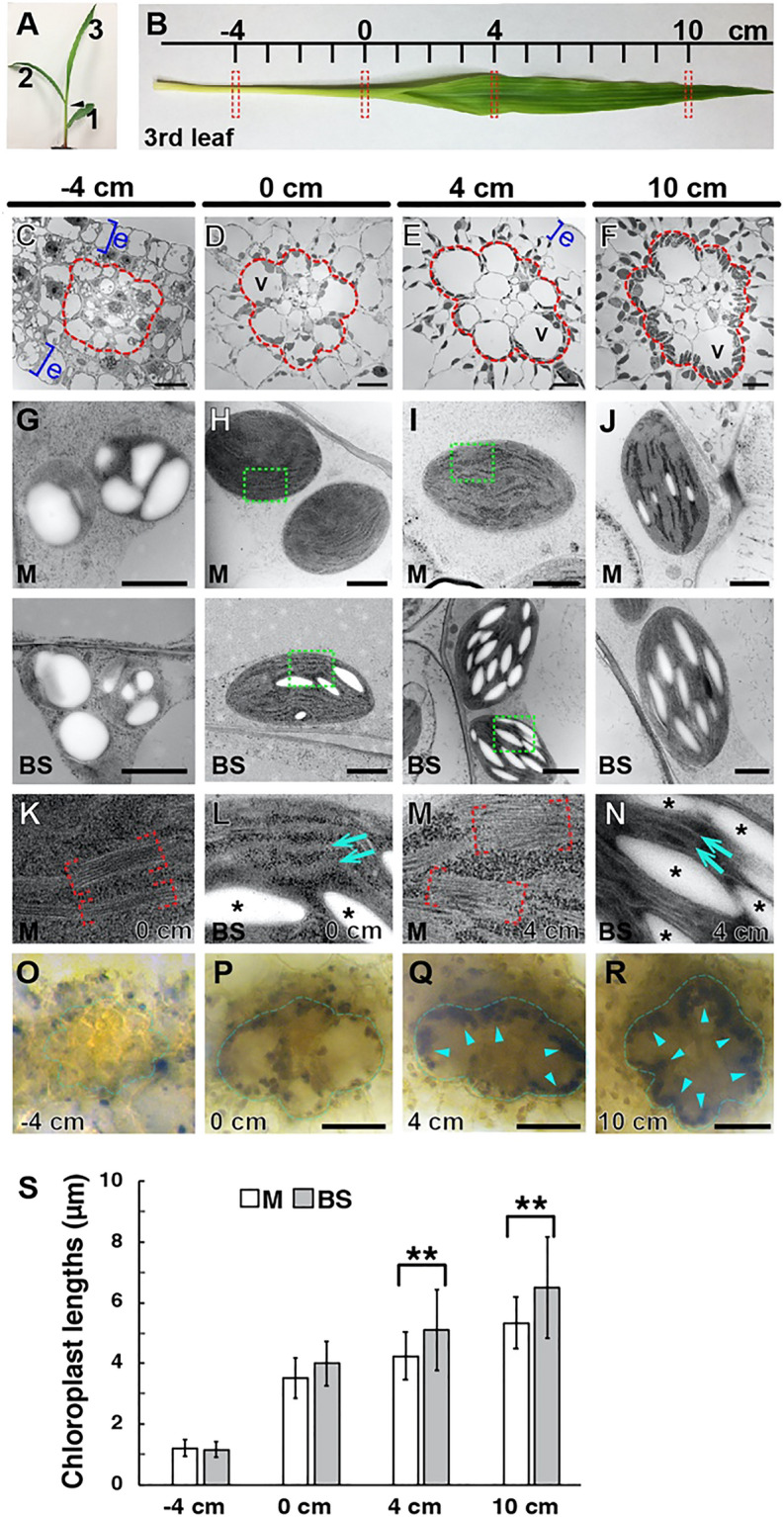
Dimorphic chloroplasts in the B73 seedling leaf. **(A)** A 9-day old maize B73 seedling. The first (1), second (2), and third (3) leaves are marked. **(B)** The four positions (−4, 0, 4, 10 cm) of the third leaf were isolated for microscopy analysis (dashed rectangles). **(C–F)** Low-magnification TEM images of the four segments in (**B**). The vascular bundle and BS cells are enclosed in the red outline in each panel. Blue brackets indicate epidermis (e) layers. *V* vacuole. Scale bars: 10 µm. **(G–J)** TEM photos of chloroplasts in M cells (upper row) and BS cells (lower row) in the four locations along the leaf. Scale bars: 1.0 µm. **(K–N)** Higher magnification micrographs of the boxed areas in **(H)** and **(I)** to show grana stacks in M chloroplasts (red brackets in **K** and **M**) and stroma lamellae in BS chloroplasts (arrows in **L** and **N**). Starch particles are denoted with asterisks. **(O–R)** Starch granules after iodine staining. Starch accumulation in BS cells (outlined with blue lines) is discerned in the 4- and 10-cm samples (blue arrowheads). Scale bars: 50 µm. **(S)** Lengths of M (white bars) and BS (grey bars) chloroplasts in TEM micrographs. Longer axes of chloroplasts (n =  ~ 25 for each stage) were measured. Error bars correspond to standard deviations (SD) (**, p < 0.01; Student’s t-test).

We examined chloroplast structures in the maize leaf with TEM and electron tomography (ET) to characterize the ultrastructural dynamics involved in the C4 differentiation. The four locations along the developmental gradient of third leaves of 9-day-old maize seedlings were selected as defined in Li et al*.* (2010) (Fig. 1A). The location of leaf 2 ligule in leaf 3 was set as the reference point (0 cm), and maize leaves were cut perpendicular to the leaf axis at -4 cm, 0 cm, 4 cm, and 10 cm (Fig. 1B). Thin cross sections (0.2–0.3 mm) were isolated and preserved by high-pressure freezing and freeze-substitution for ET analyses. For consistency in comparing the four stages among genotypes, our examination was restricted to minor veins because they outnumber midveins and intermediate veins by 7 to tenfold^28^. We examined intermediate veins in the −4-cm samples, because minor veins do not extend to the young leaf tissue, and the cell organization of intermediate veins is more similar to that of minor veins than that of midveins. Intermediate and minor veins correspond to rank 1 and rank 2 intermediate veins, respectively, according to the classification by Sedelnikova et al*.*^5^.

We were able to discern the M and BS cells in the −4-cm cross section but the concentric arrangement indicative of Kranz anatomy was not apparent (Fig. 1C). In 0-cm cross sections, however, the vascular bundle, BS cells, and M cells outside the BS layer were visible (Fig. 1D). Proplastids in M and BS cells at −4 cm were indistinguishable: They were round with average diameters of 1.5–1.6 µm, and starch particles occupied most of their stroma (Fig. 1G,O) as reported in Majeran et al*.*^14^ Chloroplasts of the two cell types in the 0-cm zone were larger and had more thylakoids than those of the −4-cm zone. M chloroplasts had grana stacks but few starch particles, whereas BS chloroplasts had starch particles, but grana stacks were rare, displaying signs of differentiation (Fig. 1H,K,L). The differing thylakoid morphologies of the M and BS cells in the 0-cm sections indicate that the two cell types express different sets of chloroplast proteins.

The chloroplast dimorphism of Kranz anatomy was clearly observed in 4-cm zone chloroplasts, (Fig. 1G–J). Grana stacks were abundant in M chloroplasts, but thylakoids were mostly unstacked in BS chloroplasts (Fig. 1I,M,N). Thylakoid architectures in the two cell types in the 4-cm sections persisted in 10-cm sections (Fig. 1J). When starch particles were stained with iodine, darkly stained particles accumulated in BS cells of leaf sections from 4-cm and 10-cm positions (Fig. 1O–R). The starch accumulation correlated with the extent of chloroplast development (Fig. 1K–N). Chloroplasts were ovoid in both cell types, but BS chloroplasts were more elongated than M chloroplasts (Fig. 1S).

**Figure 2:**
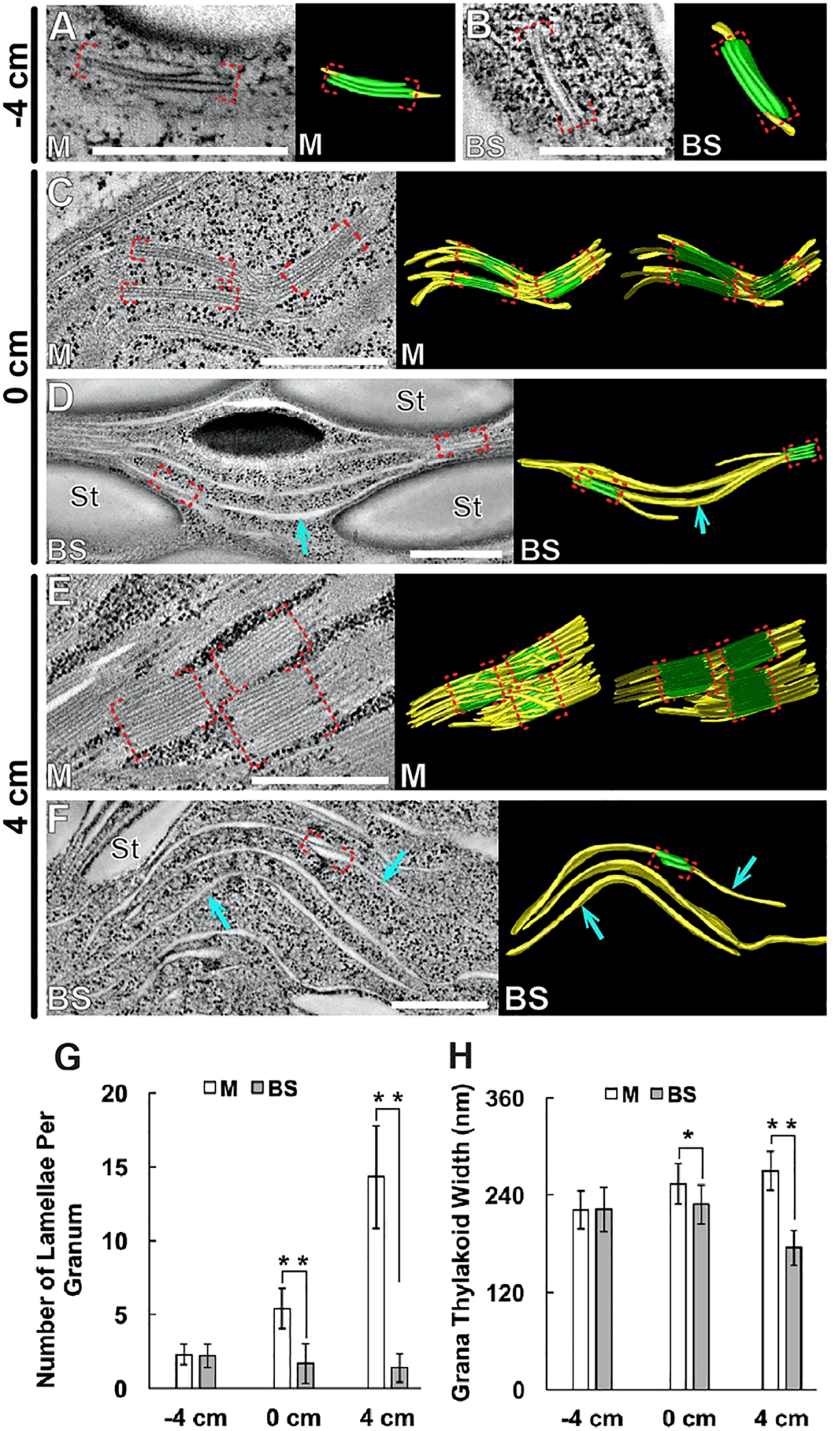
ET analysis of thylakoid assembly in chloroplasts of the maize leaf. **(A–F)** Tomographic slice images of thylakoids in M and BS chloroplasts (left) and 3D models (right) in (**A**,**B**) −4-cm, (**C,D**) 0-cm, and (**E,F**) 4-cm sections. Stacked and unstacked regions of thylakoids are colored in green and yellow, respectively in 3D models. Grana stacks were highlighted with red brackets, unstacked stroma lamellae are marked with blue arrows in (**D,F)**. In panels **(C,E)**, the thylakoid models were clipped to reveal their stack architectures. Scale bars: 500 nm. **(G)** Average numbers of disks per granum in M and BS chloroplasts (n = 10 per cell type and stage). Error bars depict standard deviations (SDs) (**, p < 0.01; ns, not significant; Student’s t-test). **(H)** Average widths of grana stacks in M and BS chloroplasts (n = 10 per cell type and stage). Error bars depict SDs (*, p < 0.05; **, p < 0.01; Student’s t-test).

To delineate thylakoid assembly more accurately, we carried out ET analysis. At −4 cm, plastids had simple thylakoid networks, and grana stacks consisted of two or three layers in both cell types (Fig. 2A,B). In M chloroplasts in 0-cm sections, each granum had approximately five layers on the average, and grana stacks densely populated the stroma (Fig. 2C). Thylakoids in BS chloroplasts of the same stage had extended stroma lamellae and grana stacks consisting of 2–4 disks (Fig. 2D). M chloroplasts had thylakoids clearly distinct from those of BS chloroplasts at 4 cm (Fig. 2E,F). Grana stacks in M chloroplasts often had more than 10 disks interconnected by tubules surrounding the stacks. By contrast, thylakoids in BS chloroplasts were composed mostly of unstacked lamellae. Grana stacks consisted of only two or three layers in BS chloroplasts at 4 cm and they were smaller than those of younger BS cells (Fig. 2G,H). These results indicate that stroma lamellae expanded, and grana stacks shrank in BS chloroplasts during leaf maturation.

### Localization of chloroplast proteins in M and BS cells by immunofluorescence

**Figure 3:**
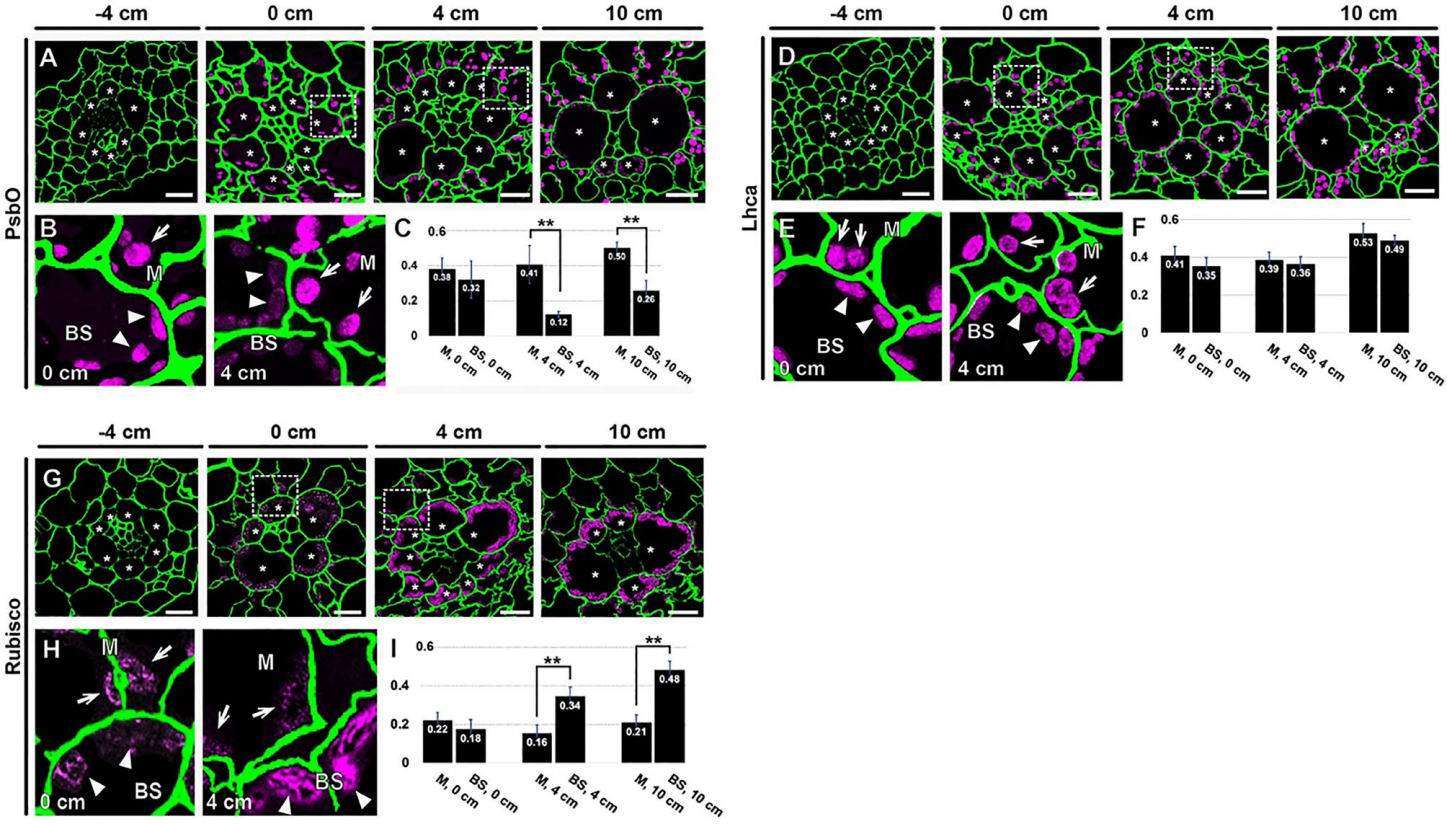
Immunofluorescence localization of chloroplast proteins in M and BS cells at the four leaf development stages. Localization of (**A–C**) PsbO, (**D–F**) Lhca, and (**G–I**) Rubisco large subunit was detected. For each protein, low magnification micrographs (upper images) and higher magnification micrographs of the boxed areas in 0 and 4 cm sections (lower left images) are shown. Cell walls (green) were stained to illustrate Kranz anatomy. BS cells are marked with asterisks in the low magnification images. M and BS chloroplasts are indicated with arrows and arrowheads, respectively, in high magnification images. M chloroplasts are more strongly stained by the PsbO in 4-cm section (**B**). The opposite is true Rubisco in 4-cm sections **(H)**. Scale bars: 10 µm. Inset graphs (**C,F,I**) plot fluorescence intensities from M and BS chloroplasts in 0- 4-, and 10-cm sections. Chloroplast fluorescence values were normalized with cell wall fluorescence intensity in each micrograph. Error bars depict standard SDs (**, p < 0.01; Student t-test).

Our ET results indicated that the chloroplast structures in M and BS deviated from each other in the 0-cm location and were fully differentiated by 4 cm (Figs. 1 and 2). We performed immunofluorescence localization of subunits in the thylakoid membrane protein complexes and of Rubisco to compare the structural dimorphism with macromolecular compositions of the chloroplasts. We utilized antibodies against PsbO (a subunit of PSII), Lhca (a subunit of the light harvesting complex of PSI), and the large subunit of Rubisco. None of these proteins were detected in the −4-cm samples (Fig. 3A,D,G). In 0-cm samples, we were able to image fluorescence from both M and BS chloroplasts, but significant differences between the two cell types were not detected (Fig. 3B,E,H).

Differential enrichment of PsbO and the Rubisco large subunit in either M and BS chloroplasts was clearly seen in 4-cm samples (Fig. 3B,H). PSII and its light harvesting complexes contribute to the formation of grana stacks. Consistent with the proliferation and removal of grana stacks in M and BS chloroplasts, respectively, PsbO accumulated in M chloroplasts in the 4-cm regions (Fig. 3B,C). Lhca-specific fluorescence, indicative of PSI, was detected in both M and BS chloroplasts throughout the leaf developmental gradient (Fig. 3E,F). The Rubisco large subunit was detected primarily in BS chloroplasts in the 4-cm section (Fig. 3H,I). The cell-type specific accumulation of Rubsico suggests that the Calvin cycle operates primarily in BS chloroplasts in the 4-cm leaf cells.

Chlorophyll fluorescence from PSI is enriched in the spectral window above 700 nm, whereas that from PSII dominates the window below 700 nm; this feature has been utilized to visualize dimorphic chloroplasts in C4 plants^40,41^. We therefore tested whether PSII fluorescence is weaker from BS in the 4-cm leaf samples. PSII intensities were similar in M and BS cells in the −4- and 0-cm sections (Supplemental Fig. S1A,B). By contrast, PSII-specific fluorescence was lower in BS cells than M cells in 4-and 10-cm sections (Supplemental Fig. S1C,D). There were no differences in the fluorescence detection range of 720–800 nm (i.e., PSI) in chloroplasts from BS cells compared to those from M cells at any stage. Thus, the PSI- and PSII-specific chlorophyll fluorescence imaging agrees with the immunofluorescence localization data.

### PD at the M-BS boundary acquire sphincters and cytoplasmic sleeves

**Figure 4:**
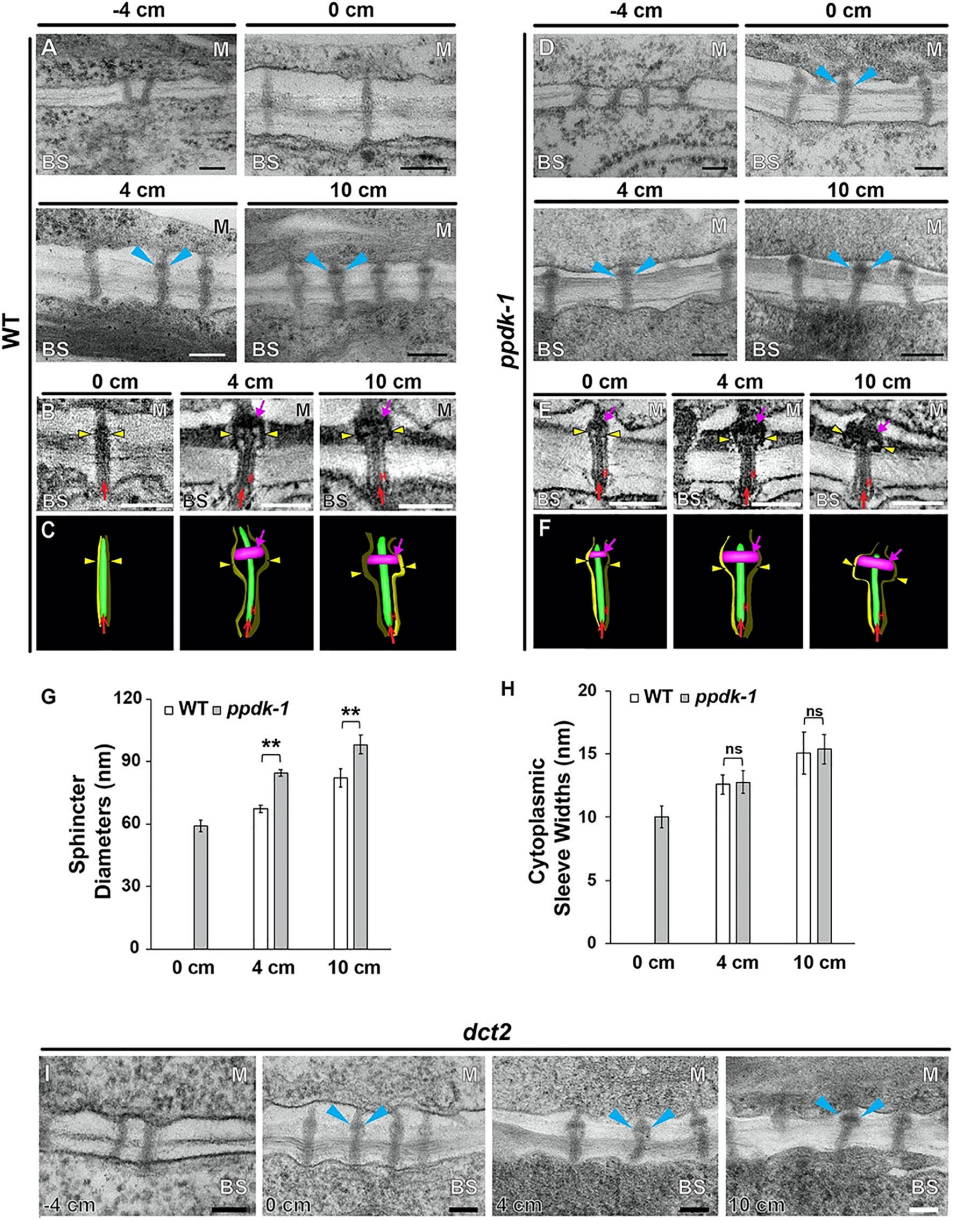
Ultrastructure of PD connecting M and BS cells. **(A)** TEM micrographs of PD at the M-BS interface in wild-type B73 (WT) leaves. PD sphincters are marked with arrowheads. Scale bars, 100 nm. **(B,C)** PD and the plasma membrane at the M-BS interface as observed in (**B**) ET slice images and (**C**) 3D models. Sphincters, desmotubules, plasma membrane, and cytoplasmic sleeves are marked with magenta arrows, red arrows, yellow arrowheads, and red “H”s respectively. Scale bars, 150 nm. **(D–F)** TEM micrographs of PD at the M-BS interface in (**D**) *ppdk-1* leaves, (**E**) ET slice images, and (**F**) 3D models. Sphincters, desmotubules, plasma membrane, and cytoplasmic sleeves are marked with magenta arrows, red arrows, yellow arrowheads, and red “H”s respectively. (**G,H**) Morphometric comparisons of PD at the M-BS interface in WT (white bars) and *ppdk-1* (gray bars) leaves. **(G)** Sphincter diameters and **(H)** cytoplasmic sleeve widths were measured from tomograms at the three stages. Approximately 20 PD from three different plants were examined for each genotype and each stage. Error bars depict SDs (**, p < 0.01; ns: not significant; Student’s t-test). **(I)** TEM micrographs of PD at the M-BS interface in *dct2* leaves. PD sphincters were discerned in 0-cm sections from the *ppdk-1* and *dct2* mutant leaves. Sphincters are indicated by arrowheads.

It was previously shown that PD connecting an M and BS cell pair has a collar around its desmotubule, termed a sphincter; the sphincter is located on the M side^23^. In TEM micrographs of −4-cm leaf sections, PD were simple and devoid of sphincters. Thickness of the cell wall between M and BS cells almost doubled from the −4-cm to the 0-cm section (Fig. 4 and Supplemental Fig. S2). PD in the 0-cm cell wall were longer than those in the −4-cm cell wall. But they remained simple PD lacking cytoplasmic sleeves or electron-dense collars around the desmotubule. Sphincters were observed in PD of 4-cm and 10-cm leaf samples (Fig. 4A). When visualized with ET, the doughnut-shaped sphincters appear to surround the desmotubule and press the plasma membrane outward, enlarging the neck region. Cytoplasmic sleeves were observed in the BS half of PD in 4-cm sections as were sphincters in the M side (Fig. 4B,C). These structural changes in PD correlated with chloroplast differentiation between 0-cm and 4-cm.

We then examined PD that join the same cell types to determine whether they undergo changes similar to those observed for PD that join M and BS cells. The sphincters were discerned in PD that connect two M cells in 4- and 10-cm sections. However, sphincters were not observed in PD connecting two BS cells (Supplemental Fig. S2). The PD connecting two M cell walls had sphincter collars on both sides as previously observed^28^, indicating that the attachment is derived from the M cell. The thickness of the BS cell wall increased from −4 to 4 cm, but the M cell wall stopped thickening at 0 cm (Supplemental Fig. S2).

### PD maturation at the M-BS boundary occurs in younger leaf zones of C4 mutant leaves

Because biogenesis of dimorphic chloroplasts is essential for C4 photosynthesis, the PD remodeling might be related to the C4 cycle. To test this notion, we compared PD in *ppdk-1, a* null allele of C4 PPDK in maize and *dct2* (*dct2-1* in Weissman et al. 2016^7^) of which malate transport into BS chloroplasts is inhibited. PD developed more rapidly in the maize mutant lines than in wild-type plants (Fig. 4D–I). PD in the *ppdk-1* and the *dct2* mutant leaves elongated as the cell wall thickened from −4 to 0 cm. ET imaging revealed that sphincter assembly had begun before 0 cm in the mutant leaves (Fig. 4D,I). The PD in the 0-cm mutant leaves had acquired cytoplasmic sleeves. These morphological changes were not detected in wild-type PDs until 4 cm (Fig. 4A–C). Sphincter sizes were larger in *ppdk-1* PD than wild-type PD at the same leaf positions (Fig. 4G) probably as sphincters developed earlier in the mutant PD. However, widths of cytoplasmic sleeves did not differ between mutant and wild-type plants (Fig. 4H). The accelerated PD changes were observed in the *dct2* mutant (Fig. 4I) and two other *ppdk* mutant alleles*, ppdk-2 and ppdk-3* (Supplemental Fig. S3).

Maize plants homozygous for *ppdk-1 and dct2* mutations are seedling lethal, but their development from germination through 9 days of growth (i.e., the time of sampling) was relatively normal (Supplemental Fig. S4). The Kranz anatomy was not affected by the deficiency in C4 pathways, although their leaves were pale green. Chloroplast proteins were differentially enriched in M and BS chloroplasts between the 0-cm and 4-cm stages in the mutant leaves as in the wild-type leaf (Supplemental Fig. S5). Transcriptomic datasets from *ppdk-1* and *dct2* mutant leaves are available and we performed gene expression correlation test using the RNA-seq datasets^7,8^. Correlation coefficients of genes were calculated from their expression profiles at the four leaf positions in wild type and the mutant lines. The mutations did not cause large scale transcriptomic alterations in mutant leaves (Supplemental Fig. S4).

### Reduced symplastic dye movement at the M-BS boundary correlated with PD maturation

**Figure 5:**
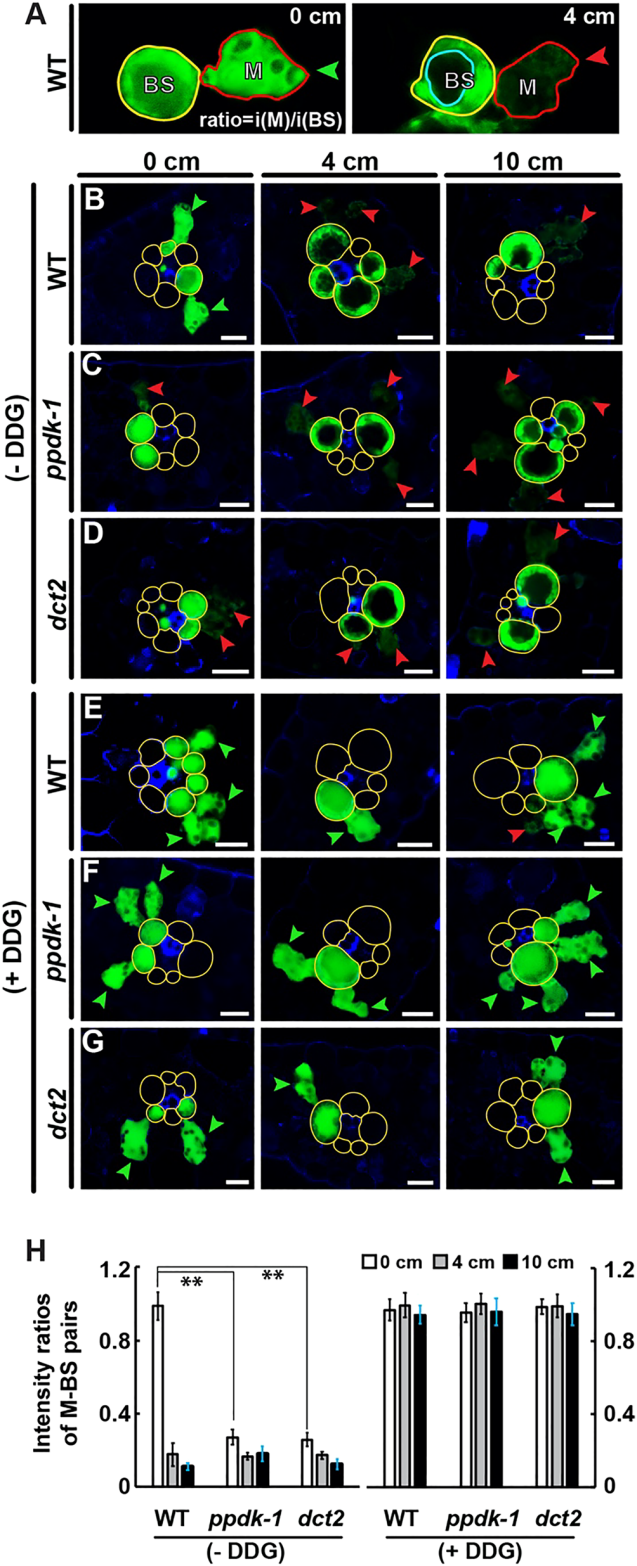
CFDA movement assay. **(A)** Confocal laser scanning micrographs of CFDA fluorescence (green) in WT leaf cross sections (0 and 4 cm). Typical M-BS pairs at 0 cm (left) and 4 cm (right) in WT leaf cross sections emit CFDA fluorescence. Green arrowhead marks fluorescence in M cell comparable to that in BS cell. Red arrowheads indicate faint fluorescence in M cell. Light blue compartment indicates central vacuole. **(B–G)** WT, *ppdk-1*, and *dct2* leaves (**B–D**) without DDG or (**E–G**) with DDG. M-BS units in which the M cells display fluorescence comparable to (less than 30% reduction) the BS cell are denoted with green arrowheads. Red arrowheads mark M cells in which fluorescence is weaker (less than 30% that in BS cell). Note that M cells in the 0-cm *ppdk-1* and *dct2* sections do not contain dye. BS cell walls are highlighted with yellow lines. Scale bars: 25 µm. **(H)** CFDA fluorescence ratios in M-BS units. The fluorescence intensities from the cytosols of M and BS cells were quantified to determine ratios. Central vacuoles were excluded when intensities were calculated (**, p < 0.01; Student’s t-test).

To investigate consequences of the PD remodeling on transport capacity, we carried out a carboxyfluorescein-diacetate (CFDA) movement assay. After leaves were fed with CFDA solution from their base, cross sections (1–2 mm thick) at 0, 4, and 10 cm were dissected. In 0-cm sections of wild-type leaves, both M and BS cells had CFDA fluorescence (Fig. 5A,B). By contrast, the fluorescence was highly confined to BS cells in 4- and 10-cm samples (Fig. 5A,B). These results indicate that PD permeability is reduced in the more mature leaf tissues. In *ppdk-1* and *dct2* mutant leaves, CFDA was limited to BS cells in all three locations (Fig. 5C,D). CFDA was detected in M cells of wild-type and mutant leaves in all three stages when the dye solution also contained 2-deoxy-d-glucose (DDG), a callose synthase inhibitor (Fig. 5E–G), indicating that callose synthesis is required for the reduction in dye transport. When we calculated the ratios of fluorescence intensities in M-BS cell pairs, the largest differences were detected in 0-cm samples from wild-type and mutant leaves because the dye failed to diffuse into M cells from BS cells at this stage (Fig. 5B–D,H). We concluded that C4 development in the maize leaf involves a decrease in symplastic permeability between M and BS cells and that the decrease is associated with structural changes in PD.

### Suberin deposition in the BS cell wall in younger leaf zones of C4 mutant leaves

**Figure 6:**
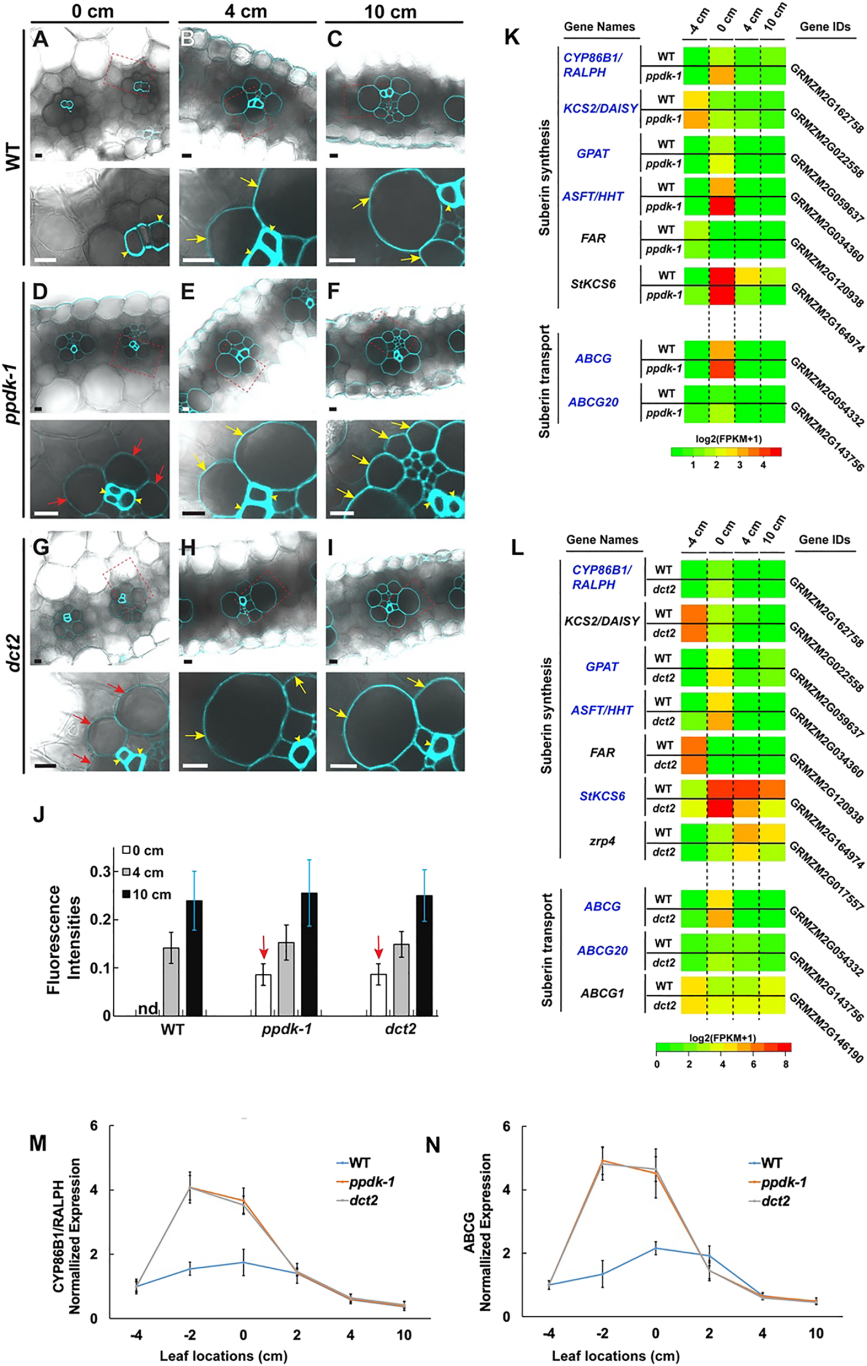
Suberin accumulation in BS cell wall. **(A–I)** Leaf sections at 0 cm, 4 cm, and 10 cm from (**A–C**) WT B73, (**D–F**) *ppdk-1*, and (**G–I**) *dct2* plants stained for suberin. In each panel, lower micrograph images correspond to higher magnifications of the boxed areas in upper micrographs. BS and xylem cell walls visualized by suberin staining are indicated with arrows (red arrows for 0-cm and yellow arrows for 4-and 10-cm images) and arrowheads, respectively. Note that BS cell walls in 0-cm *ppdk-1* and *dct2* samples stain for suberin staining **(D,G)**, whereas BS cell walls in 0-cm WT sample do not **(A)**. Scale bars: 10 µm. **(J)** Fluorescence intensities of BS cell walls in WT, *ppdk-1*, and *dct2* leaf 0-cm (white bars), 4-cm (gray bars), and 10-cm (black bars) sections. Fluorescence values of BS cell walls were normalized to those of adjacent xylem walls. **(K,L)** Heat maps illustrating changes in transcript levels of genes involved in suberin synthesis or transport in *ppdk-1* **(K)** and *dct2* **(L)** leaves. All genes with FPKM values higher than 1 were included in the heat maps (Supplemental Fig. S7). Genes transcribed more actively in the mutant than WT in 0-cm are in blue letters. **(M,N)** qRT-PCR-based quantification of **(M)** *CYP86B1* and **(N)** *ABCG* in six locations in the maize leaf. *GAPDH* was used as the reference gene. Error bars indicate SDs.

The BS cell wall accumulates suberin, and the hydrophobic stratum blocks apoplastic diffusion of photosynthetic metabolites^31^. Since this barrier could restrict solute exchange between M and BS exclusively through PD, we assayed for suberin deposition in the cell wall of the four-leaf zones. The BS cell wall in 0-cm cross sections did not exhibit fluorescence. Only xylem cells in the vascular bundle were stained in the sections (Fig. 6A–C). BS cell walls in 4-cm sections were positive for suberin staining, and the fluorescent outline visualized the BS cell layer outside the vascular bundle (Fig. 6A–C). By contrast, suberin was clearly observed in BS cell walls of 0-cm leaf sections from *ppdk-1* and *dct2* plants (Fig. 6D–I). The staining became stronger at later leaf developmental stages of wild-type as well as the mutants (Fig. 6J).

A recent microscopy study revealed that suberin deposition in the *Arabidopsis* root endodermal cell wall involves the accumulation of vesicular tubules in the gap between the plasma membrane and the secondary cell wall^42^. The complex membranous carriers appeared in non-endodermal cells where suberization was ectopically induced. We examined TEM samples from wild-type and the C4 mutant leaves (−4, −2, 0, 2, and 4 cm sections) to determine whether similar apoplastic membrane tubules are seen in the suberizing BS cell wall (Supplemental Fig. S6). In wild-type leaves, round plasma membrane ingrowths bulging with vesicular tubules appeared in BS cells from 0 and 2 cm sections. But in 4 cm sections where BS suberization is evident, we did not find such structures. By contrast, we detected extracellular pockets of vesicular tubules in the BS cell wall from −2 cm sections of *ppdk-1* and *dct2* mutant leaves. These extracellular carriers disappeared in 0 cm sections in the mutants, in agreement with their early suberization (Fig. 6A–I).

We examined expression levels of genes that play roles in suberin synthesis or export in the RNA-seq datasets from *ppdk-1* and *dct2*^7,8^. Maize genes associated with cell wall suberization were identified with the MapMan tool for functional classification (http://gabi.rzpd.de/ projects/MapMan/). Most of the identified genes exhibited strong transcriptional activities in the −4- or 0-cm zones (Fig. 6K,L). When transcript levels in wild-type and the mutant lines were compared, suberin-related genes were more upregulated in the *ppdk-1* (6 out of 8) and *dct2* (6 out of 10) leaves than wild-type leaves, in agreement with the suberin staining results. To evaluate temporal expression profiles more accurately, we carried out qRT-PCR analysis of *CYP68B* (also known as *RALPH;* GRMZM2G162758) and *ABCG* (GRMZM2G054332) genes in six leaf regions. Transcripts from the two genes were most abundant in -2-cm specimens of *ppdk-1* and *dct2* leaves and in 0-cm specimens of wild type leaves (Fig. 6M,N), indicating that the suberin-related genes were more strongly upregulated in the zone (−2 to 2 cm zone) encompassing the 0 cm leaf segment where we observed suberin staining in the mutant BS cell wall. When we evaluated maize genes that the MapMan tool identified as PD components, there were no significant differences in expression profiles in the mutant compared to wild-type leaves (Supplemental Fig. S7 and Dataset 1).

## Discussion

**Figure 7:**
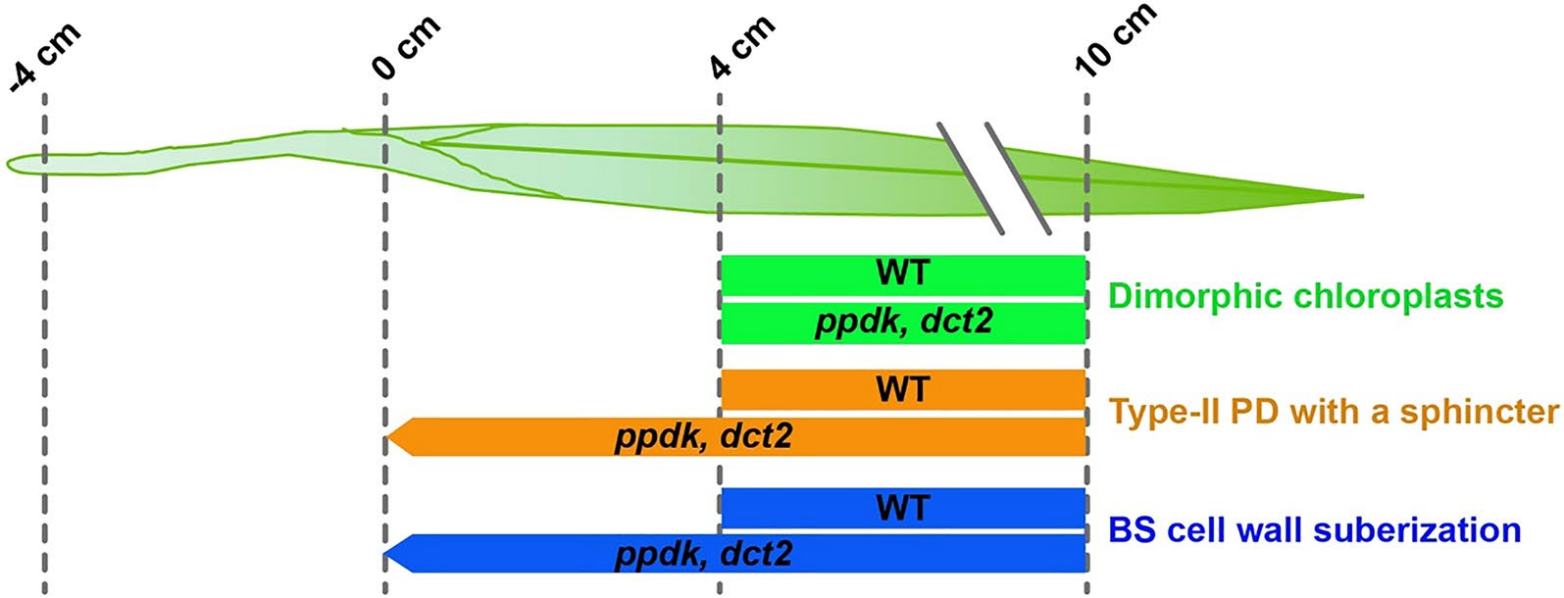
Differentiation/maturation timelines of chloroplasts, PD, and BS suberization in the maize leaf. Type-II PD refer to PD with cytoplasmic sleeves^55^. PD maturation and BS suberization are observed from the 0 cm zone of *ppdk-1* and *dct2* mutant leaves.

The timeline of chloroplast diversification in M and BS cells included assembly of thylakoids with distinct architectures, and cell type-specific accumulation of chloroplast proteins at 4 cm. The 0- to 4-cm sector corresponded to the region in which PD across M and BS acquire sphincters and cytoplasmic sleeves. Symplastic dye movement was impeded after the structural changes of PD in a callose-dependent manner, implying that the mature PD can be gated. The BS cell wall became suberized in parallel with the PD changes. In *ppdk-1* and *dct2* mutant leaves, PD remodeling and suberin deposition were finished in the 0-cm zone, implying that genes for the modifications in the M-BS interface are activated earlier if the C4 cycle is disrupted (Fig. 7).

Our TEM and ET results from the wild type agree with previous structural, transcriptomic, and proteomic studies of maize C4 leaf development. Using cell-type specific RNA-seq, Tausta et al*.* demonstrated that genes involved in the C4 cycle and PSII-related genes begin to be differentially expressed in M cells and BS cells from the sink to source transition zone^43^. The transition zone is close to our 0-cm position, where we discerned varying ultrastructural features in chloroplasts. The 0-cm position is the zone at which light availability drastically increases for photosynthesis because leaf 3 cells are wrapped inside the older leaves below this position. In a proteomic analysis of maize BS cells and vascular bundle samples, normalized levels of PSII subunits decreased in samples 4.5 cm from the leaf 3 ligule, suggesting that cyclic electron transport becomes dominant in the bundle sheath at this location^14^. This location overlaps with the 0-cm zone in our classification, and we found that grana stacks were scarcer in BS chloroplasts than in M chloroplasts in this section. We detected structural dimorphism of the M and BS chloroplasts in 0-cm sections. Fluorescence microscopy-based protein localization methods failed to distinguish M and BS chloroplasts in 0-cm sections, however; probably because differences in protein compositions were too small for detection by immunolabeling.

De Bellis et al. (2021) reported that extracellular membrane structures accumulate in suberizing *Arabidopsis* root endodermal cells^42^. The root endodermis and BS in the leaf are anatomically equivalent in that they are cell layers encircling the vascular tissue^44^. They also acquire a diffusion barrier made of suberin at maturity. SCARECROW is a transcription factor that controls the root endodermis differentiation. *SCARECROW* is expressed in BS cells and its mutation affected the BS organization, suggesting that genetic programs for the root endodermis and BS cells in the leaf are conserved^9,45,46^. We discerned round plasma membrane ingrowths in suberizing BS cells analogous to the apoplastic vesicular tubules in the *Arabidopsis* endodermal cells (Supplemental Fig. S6). Our data showed that the leaf BS cells and root endodermal cells export substances for the cell wall suberization via similar vehicles.

Abnormal suberin layers in the BS cell wall of mutant *Setaria viridis* leaves caused loss of CO2 from BS cells, decreasing their C4 photosynthetic efficiency^39^. The concentration of CO2 to BS chloroplasts is expected to be affected in *ppdk-1* or *dct2* leaf cells as C4 cycle is aberrant in them. The reduced CO2 partial pressure in the mutant BS cells may stimulate a regulatory mechanism that activates suberin synthesis and transport (Fig. 6). However, we did not observe a shift in the timing of cell type-specific chloroplast differentiation and protein expression in the mutant leaves (Fig. 7, Supplemental Figure S5). It is possible that the genetic program for constructing dimorphic chloroplasts may be less sensitive or slower in responding to the defects in C4 cycle.

PD structures and permeability evolve as the cells that they connect undergo differentiation^47,48^. PD transport is regulated to coordinate organ development and tissue patterning, because non-cell autonomous signaling molecules and transcription factors diffuse through PD^49–51^. A recent ET study showed that nascent PD in *Arabidopsis* root meristem cells lack cytoplasmic sleeves, but sleeves do form in PD of columella cells derived from meristem cells^52^. Contrary to the idea that molecular transport happens through the cytoplasmic sleeves, CFDA and GFP diffused among meristem cells despite the finding that PD in these cells lack gaps. Our connectivity assay data are consistent with the observation. CFDA crossed the M-BS boundary in the 0-cm leaf samples, where cytoplasmic sleeves were resolved.

Sphincter modules have been reported in PD, and they are linked to inhibition of cell-to-cell transport. In dormant shoot apical cells of birch trees, symplastic transport is blocked, and PD of the inactive cells are clogged with electron-dense collars^53^. When the shoot apical cells are activated, the collars disappear, and amounts of callose at the PD are reduced. In the maize leaf, sphincters in PD spanning the M-BS cell wall are associated with slow photosynthesis in plants grown at low temperature^54^. We demonstrated that sphincters appear earlier in the *ppdk-1* mutant leaves and that inhibition of callose synthase facilitated CFDA movement. Because sphincter and callose could obstruct cytoplasmic sleeves, these PD components may regulate permeability at the M-BS boundary. We speculate that the expedited PD modification and BS cell wall coating in the mutant leaves could be a feedback reaction to inhibition of CO_2_ concentration in their BS cells due to aberrant C4 cycle.

## Methods

### Plant material and growth conditions

*Zea mays* B73 (wild type for *ppdk-1*, *ppdk-2*, and *ppdk-3*), W22 (wild type for *dct2*) and four mutant lines (*ppdk-1*, *dct2*, *ppdk-2*, and *ppdk-3*) were grown from seeds in a growth chamber with 12 h:12 h light/dark and 31 °C light/22 °C dark cycle with light intensity of 550 µmol/m^2^/s and relative humidity of 50% as previously described^9^. The inbred and mutant lines were obtained from the maize stock center (http://maizecoop.cropsci.uiuc.edu/) and they were grown under the institutional guidelines. Four segments of maize leaf tissues were collected from leaf 3 on day 10 after planting, base (0 cm, the position of leaf 2 ligule as the 0 point), early stage (-4 cm, 4 cm below the 0 point), maturing stage (4 cm, 4 cm above the 0 point) and mature stage (10 cm, 10 cm above the 0 point, close to the leaf tip). This sampling scheme was adapted from Li et al*.* (2010)^9^. Leaf tissues were pooled from 10 seedlings for each genotype and each stage. At least three specimens from each pool were examined for morphometric comparison as well as protein localization experiments. The *dct2* mutant line was identified from the UniformMu population (https://www.maizegdb.org/ uniformmu) and ordered from Maize Stock Center (http://maizecoop.cropsci.uiuc.edu/). The mutant line was backcrossed 4 generations to W22 to get rid of the interference of other Mu insertions. Leaf samples for microscopy analyses were harvested within 2–4 h after the onset of the light period. For TEM imaging of starch particles, however, leaf samples were dissected at 2 h before the end of the light period.

### Transmission electron microscopy (TEM) and electron tomography

TEM and electron tomography analyses of PD were carried out as described in Kang (2010)^56^ and Koh et al. (2011)^20^. We examined maize leaf samples preserved by high-pressure freezing for electron tomographic characterization of three-dimensional thylakoid structures. However, we employed chemically fixed samples for determining PD structures and starch particle analysis. We had to capture TEM micrographs from more than hundreds of PD and chloroplasts for statistical comparisons of PD and chloroplasts in multiple stages from several genetic backgrounds. It was impossible to find sufficient numbers of intact PD or chloroplasts in maize leaf samples processed by HPF because of freezing damages.

Maize leaves (leaf 3) were dissected and cryofixed with an HPM100 machine (Leica Microsystems). The frozen samples were freeze substituted in 2% OsO4 in anhydrous acetone (Leica EM AFS2) at −80 °C for 24 h and slowly warmed from – 80 °C to −45 °C over 36 h. After being brought to room temperature, the samples were washed with anhydrous acetone three times, and embedded in Embed-812 resin (Electron Microscopy Sciences; Cat. No. 14120) and cured at 60 °C for 24 h. For chemical fixation, maize leaf segments were dissected in 2.5% glutaraldehyde in 0.1 M cacodylate buffer (pH 7.4) and incubated in the fixative solution overnight at 4 °C. After thorough washing (4–5 times) with 0.1 M cacodylate buffer (pH 7.4), the samples were post-fixed in 1% OsO_4_ in deionized water for 2 h at room temperature. Excess OsO_4_ was removed by washing with water (4–5 times), and the samples were dehydrated with a graduated acetone series in deionized water (from 10 to 100% acetone). Finally, they were embedded in Embed-812 resin (Electron Microscopy Sciences, Cat. No. 14120) and polymerized at 60 °C for 24 h. Ultrathin sections (90-nm thick) were collected on formvar-coated copper slot grids (Electron Microscopy Sciences; Cat. No. GS2010-Cu) and the sections were post-stained with uranyl acetate and lead citrate solutions. The sections were examined with a Hitachi 7650 TEM (Hitachi-High Technologies) operated at 80 kV.

A series of semi-thick sections (250 nm) were collected on copper grids. After post-staining and gold particle coating, tilt series were collected with a 200-kV Tecnai F20 intermediate voltage electron microscope (+ 60° to −60° at an interval of 1.5° interval around two orthogonal axes). Tomogram calculation and 3D model rendering were performed with the IMOD software package as described previously^57,58^.

For morphometric analysis of thylakoids, stroma lamellae were defined as thylakoids consisting of single layer, and grana thylakoids were defined as thylakoids with membranes in contact with opposing membranes to constitute a stack. The largest number of disks in a granum was assigned as the number of lamellae. For PD quantitative analyses, outer diameters of sphincter rings were measured. Widths of cytoplasmic sleeves corresponded to the average width of the two gaps from the desmotubule to the plasma membrane (right and left sides) in each PD.

### Immunofluorescence microscopy localization of chloroplast proteins

Maize leaves were dissected in 4% paraformaldehyde and fixed under vacuum for 90 min at room temperature for 3 h. The fixed samples were dehydrated through a graduated ethanol series (from 10 to 100% ethanol in water) and embedded in LR White resin (Electron Microscopy Sciences, Cat. No. 14383). After curing sample blocks, semi-thick sections (400 nm) were prepared with an ultramicrotome (Leica UC7, Leica Microsystems) and collected on glass slides (SuperFrost™, Thermo Scientific). After blocking with 2% non-fat milk dissolved in 0.2% PBST (phosphate buffered saline with 0.2% Tween 20) for 1 h, the sections were incubated with primary antibodies (1:400 dilution) overnight at 4 °C. Information about the PsbO antibody is in Liang et al. (2018) (5). The other three antibodies, Lhca (Cat. No. AS01 005), and Rubisco (RbcL, Rubisco large subunit, form I; Cat. No. AS03 037), were purchased from Agrisera (https://www.agrisera.com). The cell wall was stained with an antibody against (1–3)(1–4)-beta-glucan (Biosupplies Australia, Cat. 400-3). The sections were rinsed five times with the blocking buffer, then were probed with the fluorescently conjugated secondary antibodies (1:1000 dilution; anti-mouse Alexa Fluor 488 or anti-rabbit Alexa Fluor 568; Invitrogen) for 2 h at room temperature. Excess stain was removed by rinsing with 0.2% PBST and deionized water. Micrographs were captured with a Carl Zeiss PALM Inverted Microscope (https://www.zeiss.com). Immunoblot analysis of the four proteins were carried out as explained in Liang et al. (2018)^13^.

For quantifying fluorescence intensities of chloroplasts, we loaded micrographs in ImageJ (ver. 1.53d), and outlines were drawn around chloroplasts to set regions of interest (ROI). Using the “Measure” command, we acquired mean intensity values inside the chloroplast outlines. To normalize chloroplast fluorescence values in different micrographs, cell wall fluorescence intensities were calculated in each micrograph and chloroplast values were divided by the cell wall intensity values. At least 30–40 randomly chosen chloroplasts in M and BS cells (from 3 plants) in 0, 4, and 10 cm sections were examined and their averages and standard deviations were calculated in Microsoft Excel.

### Chlorophyll autofluorescence imaging

Free-hand maize leaf cross-sections were mounted in water on glass slides (SuperFrost™, Thermo Scientific), and were examined using an SP8 confocal microscope to acquire the autofluorescence images of chloroplast and cell wall. After excitation with a 638 nm laser, emission wavelengths of 650–720 nm and 720–800 nm were imaged for PSII an PSI chlorophyll autofluorescence, respectively. The cell wall was visualized using its autofluorescence (excitation—405 nm, emission—420–480 nm).

### Starch staining

Maize leaves (leaf 3) were dissected and cleared by incubating in 95% ethanol overnight. The samples were stained with iodine potassium iodide (IKI) solution (6) and excess IKI solution was washed off by rinsing in deionized water. Free-hand cross-sections were examined under bright-field under a Meiji Techno MT4310L dissecting microscope (Meiji Techno Co.) equipped with a Leica MC120 HD microscope camera.

### Carboxyfluorescein diacetate (CFDA) transport assay

Maize leaves (leaf 3) were cut at their bases and put them upright in a beaker containing CFDA solution (50 µg/mL; Sigma-Aldrich, Cat No. 21879-25MG-F) to feed the dye from their cut ends. After 1 h in the dark, three leaf segments (0 cm, 4 cm, and 10 cm) were collected by free-hand dissection. CFDA dye distribution in the leaf cross-sections was examined with a Leica TCS SP8 confocal laser-scanning microscope. We could not assess CFDA in BS-M pairs of −4 cm cross-sections (close to the cut end) consistently because CFDA infiltrated into the apoplastic space and overstained all cell types. For DDG treatment, leaf 3 samples were first put in a DDG solution (0.1 mM; Sigma-Aldrich, Cat. No. D8375-1G) for 0.5 h and then transferred to the CFDA solution supplemented with DDG (50 µg/mL of CFDA, 0.1 mM of DDG) for another 1 h in the dark. Leaf sections were imaged with excitation of 488 nm and an emission range of 493–555 nm for CFDA fluorescence. The cell wall was visualized from its autofluorescence (405 nm excitation and detection window of 410–483 nm emission). To compare CFDA intensities in M and BS cells, Cytosolic areas of M and BS cell pairs were outlined in ImageJ (ver 1.53d) and mean CFDA intensity values in the outlines were acquired using the ‘Measure” command. Fluorescence ratios of 20–3- individual M-BS cell pairs were calculated from 0-, 4- and 10-cm leaf positions and their averages and standard deviations were calculated in Microsoft Excel (ver. 16.40). The cell pairs were from three leaves for each genotype.

### Cell wall suberin staining

Free-hand cross-sections of maize leaves (leaf 3) were incubated with freshly prepared solution of 0.1% (w/v) berberine hemisulfate (≥ 95%; Sigma-Aldrich, Cat. No. B3412-10G) in lactic acid (≥ 85%; Sigma-Aldrich, Cat. No. 252476-500G) with 1 g/mL chloral hydrate (≥ 99%; Sangon Biotech, Cat. No. A600288-0250) in a Petri dish covered with aluminum foil at 65 °C for 2 h. The solution was removed using a pipette, and sections were thoroughly washed with distilled water at least three times. After removing the water and drying on filter paper, we transferred the sections to 0.5% (w/v) aniline blue (C.I. 42755, Water-soluble; Polysciences, Cat. No. 02570-25) in distilled water for 1 h in the dark at room temperature. The sections were subsequently washed with distilled water three times. After mounting the sections into a drop of water on microscope slides, they were examined with a Leica TCS SP8 confocal laser-scanning microscope with excitation at 405 nm and an emission window of 500–580 nm. BS cell walls were outlined and mean intensity values inside the narrow outlines were recorded in ImageJ (ver 1.53d). 20–30 BS cell walls from three leaf samples were examined for each stage. The staining and clearing procedures were adapted from previous publications^59,60^.

### Gene expression correlation test

Correlation of gene expression tendency in wild type B73, with *ppdk-1* or with *dct2* were assessed with publicly available datasets produced by Li et al. (2010)^9^, Zhang et al. (2018)^8^, and Weissman et al. (2016)^7^. Raw data were downloaded from SRA(Accession codes: SRA012297, PRJNA280756, PRJNA340078) and mapped to maize AGPv4 genome using bowtie2. Cufflinks was used for calculating FPKM values and the Pearson correlation of the four sections between datasets were calculated using the R programming language. Violin plots were prepared from each comparison. Sources of datasets are:

B73 from Li et al. (2010), *DCT2* (wild type control for *dct2* study) & *dct2* from Weissman et al. (2016)^7^, and *PPDK* (wild type control for *ppdk* study) & *ppdk-1* from Zhang et al. (2018)^8^.

### RNA-seq analysis of suberin synthesis genes and PD component genes

Differential expression analysis of maize genes encoding factors involved in suberin synthesis and constituents of PD were performed with published RNA-seq datasets for wild-type and *dct2* mutant leaf sections (accession number GSE67722)^7^. The same analysis for *ppdk* mutant alleles was carried out with the published dataset SRP082943^8^. We downloaded the datasets from NCBI and used the RNA-seq analysis pipelines cited in the published reports. The FPKM values of suberin synthesis genes and PD component genes are listed in Supplemental Dataset 1.

### qRT-PCR verification of transcript levels

Total RNA samples were isolated with RNeasy Plant Mini Kit (Qiagen; Cat. No. 74903), and the samples were reverse transcribed with QuantiNova reverse transcription kit (Qiagen; Cat. No. 205411). Three replicates of RNA extraction were performed at each stage for wild-type, *ppdk-1*, and *dct2* lines. qRT-PCR assays were conducted (denaturation at 95 °C, annealing/extension at 60 °C, 40 cycles) to estimate amounts of transcripts from the selected genes using a CFX96 real-time PCR detection system (Bio-Rad). Each reaction mix (10 µL) contained 5 μL SsoAdvanced™ Universal SYBR Green Supermix (Bio-Rad; Cat. No. 172–5272), 1 µL of cDNA template (75 ng/µL), 0.5 µL of forward primer (500 nM) and 0.5 µL of reverse primer (500 nM). The primer sequences for qRT-PCR are in Supplemental Table S1.

## Supplementary Information

Supplementary Information 1.

Supplementary Information 2.
