# Supplementary material for: Accelerated remodeling of the mesophyll-bundle sheath interface in the maize C4 cycle mutant leaves
Source: Sci Rep. 2022 Mar 23;12:5057. doi: 10.1038/s41598-022-09135-7 (PMC8943126; doi:10.1038/s41598-022-09135-7)
Supplement: Supplementary file 1 — Supplementary Information 1. [file 41598_2022_9135_MOESM1_ESM.docx]

Supplementary figures and table for

**Accelerated remodeling of the mesophyll-bundle sheath interface in the maize C4 cycle mutant leaves**

Peng Gao, Pengfei Wang, Baijuan Du, Pinghua Li, and Byung-Ho Kang1

^1^Author for correspondence: Byung-Ho Kang ([bkang@cuhk.edu.hk](mailto:bkang@cuhk.edu.hk), 852-3943-6101)

**This PDF file includes:**

Supplementary Figures S1 to S7

Supplementary Table S1

**Supplementary Figures**


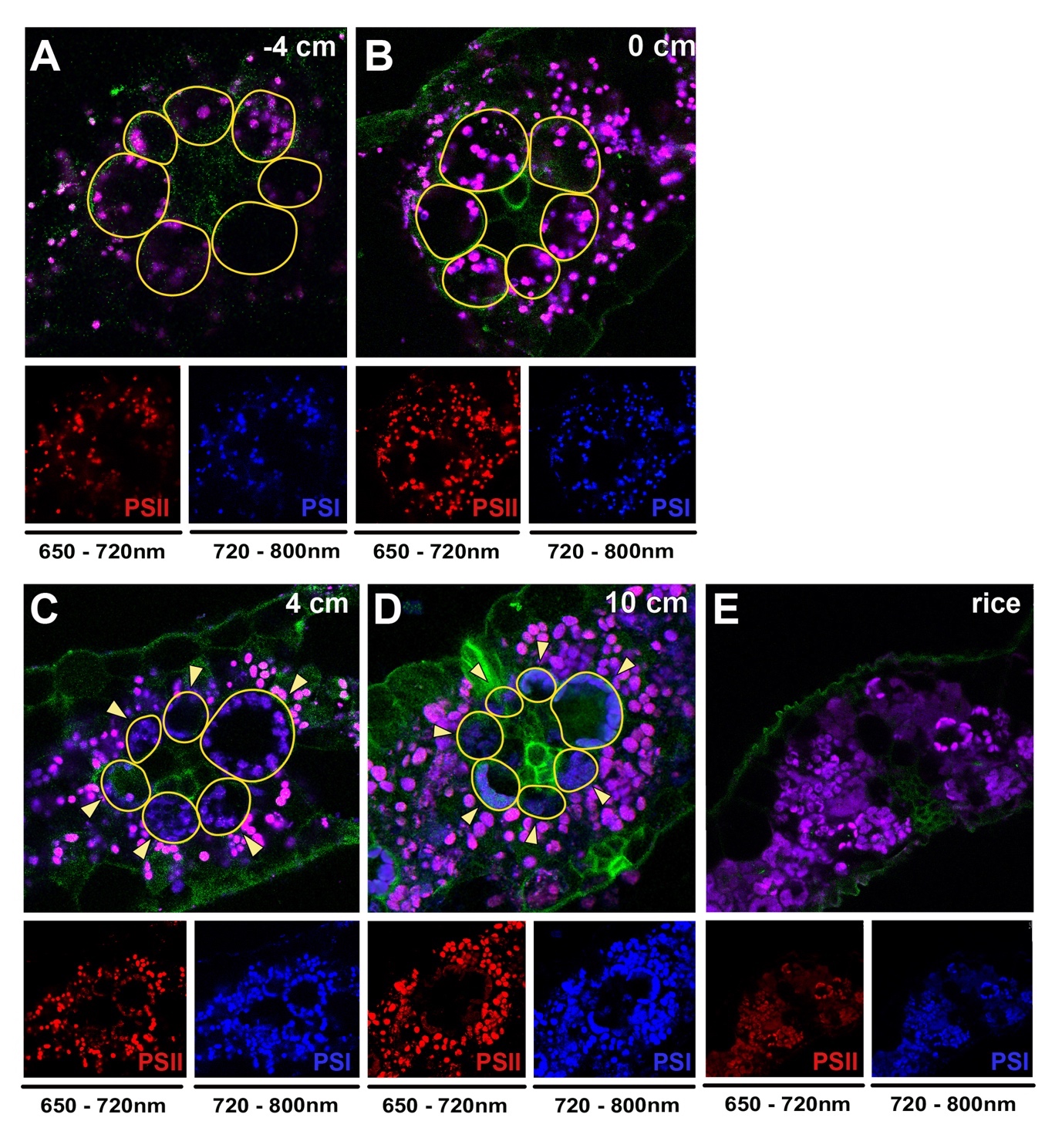


**Supplementary Figure S1.** Distribution of photosystem (PS) II and PSI in the maize leaf.

**(A-D)** Confocal laser scanning micrographs showing cross-sections of maize leaves at -4 cm (A), 0 cm (B), 4 cm (C), and 10 cm (D). Micrographs from detection window of 650-720 nm (red, PSII) and window from 720-800 nm (blue, PSI) are provided below in each panel. Bundle sheath cells surrounding the vascular bundle are highlighted with yellow lines. In mesophyll chloroplasts with both PSI and PSII, autofluorescence from the two emission ranges overlap (magenta), whereas PSII-specific autofluorescence is reduced in bundle sheath chloroplasts in 4-cm and 10-cm sections (arrowheads in **C** and **D**). Scale bars: 25 µm.

**(E)** A rice leaf section as a control for C3 photosynthesis with a single type of chloroplasts. PSII- and PSI-fluorescence overlap (magenta).

**
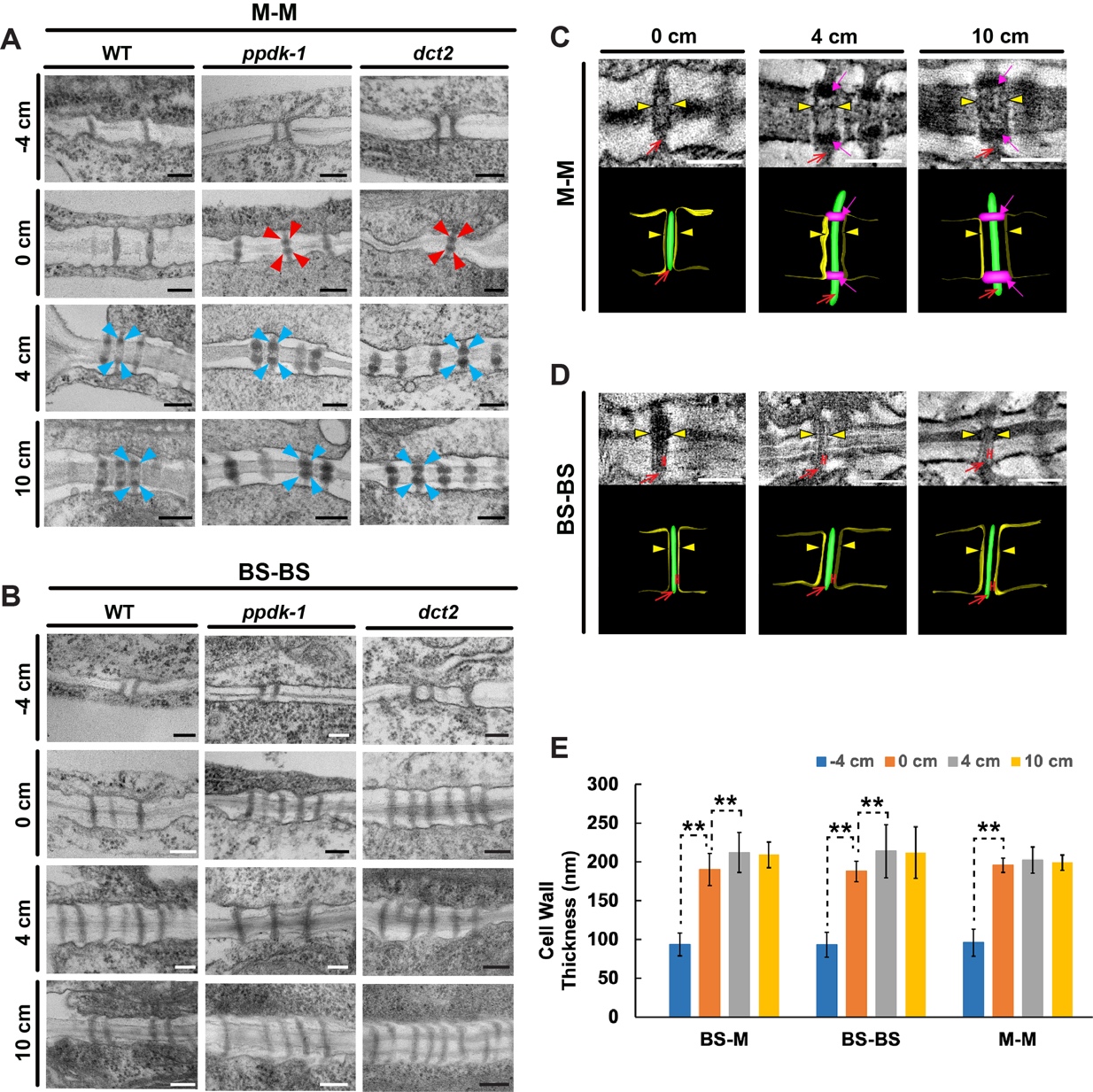
**

**Supplementary Figure S2.** TEM images of PD in leaf samples of wild-type (WT) B73, *ppdk-1,* and *dct2* maize lines.

**(A and B)** TEM images of PD between mesophyll (M) cells (A) and between bundle sheath (BS) cells (B). Sphincters are marked with arrowheads. PD of 0 cm sections in the two mutant lines have sphincters (red arrowheads). Scale bars: 100 nm.

**(C and D)** Electron tomography slice images of PD and their 3D models in M-M (C) and in BS-BS (D) cell walls in WT leaves. Sphincters (magenta), desmotubules (green), plasma membrane (yellow), and cytoplasmic sleeves were marked with magenta arrows, red arrows, yellow arrowheads, and red “H”s, respectively. PD in the BS-BS cell walls lack sphincters (D). Scale bars, 150 nm.

**(E)** Thickness of the cell walls between BS-M, BS-BS, and M-M cells of WT leaves at the four developmental stages. Thickness was measured from TEM micrographs (-4 cm, blue bars; 0 cm, orange bars; 4 cm, gray bars; and 10 cm, dark yellow bars). Cell walls of 10 cells from three leaf samples (n=30) were measured for each bar in the graph. Error bars are standard deviations (******, p<0.01 by Student’s t-test).

**
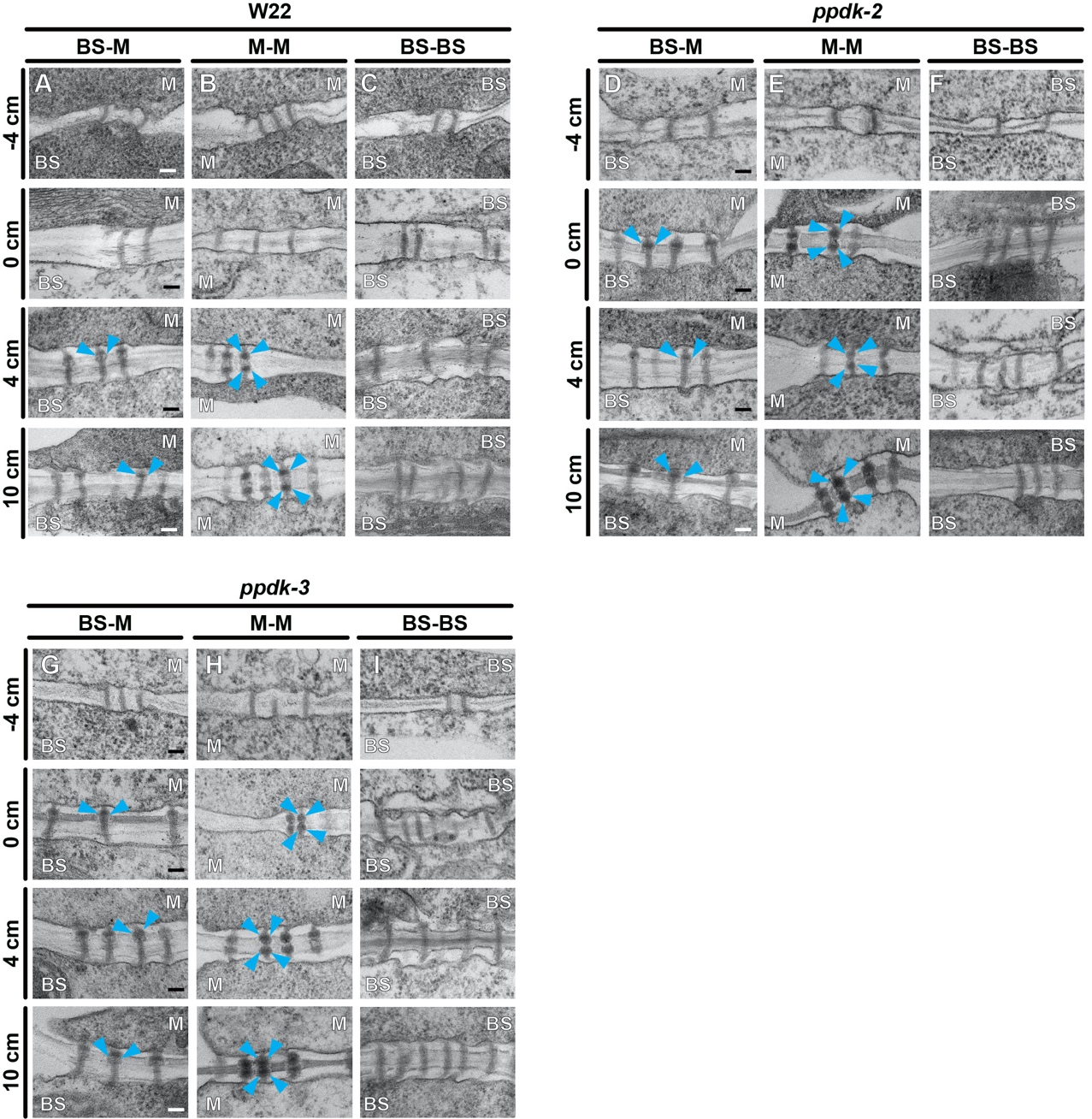
**

**Supplementary Figure S3.** TEM micrographs of PD in the maize leaf samples from wild type W22 **(A-C)**, *ppdk-2* **(D-F)** *and ppdk-3* **(G-I)** mutant alleles.

PD in cell walls between bundle sheath (BS)-mesophyll (M), M-M, and BS-BS are shown. Sphincters are marked with blue arrowheads on the M side of the cell wall. Scale bars: 100 nm.


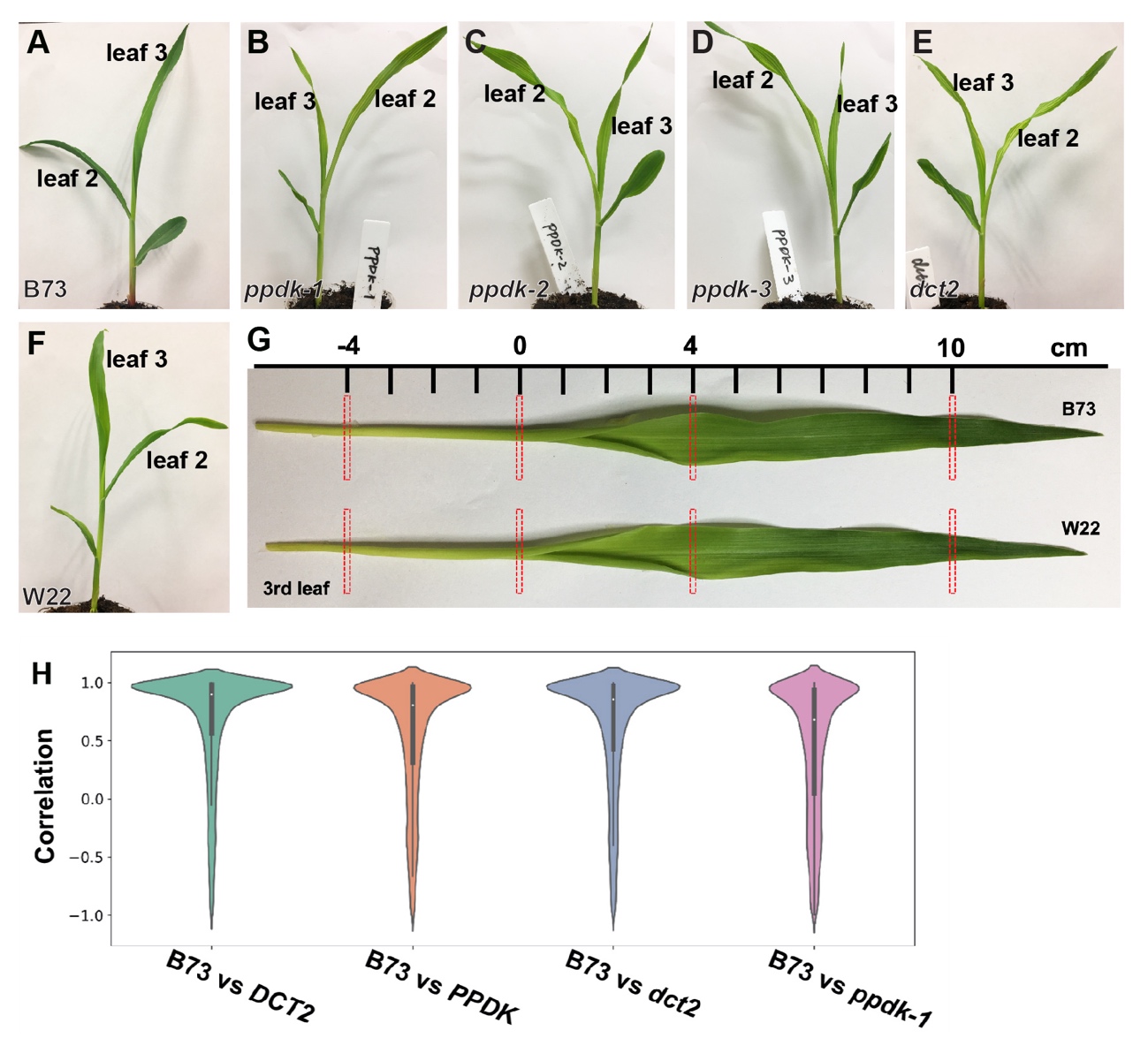


**Supplementary Figure S4.** Photographs of 9-day-old maize seedlings and gene expression correlation in wild type and mutant leaves.

**(A)** wild-type (WT) B73, **(B)** *ppdk-1*, **(C)** *ppdk-2*, **(D)** *ppdk-3*, **(E)** *dct2,* and **(F)** W22 seedlings grown under conditions described in **Methods**. Leaf 2 and leaf 3 are marked in the photos. **(G)** The four positions (-4, 0, 4, 10 cm) for developmental stages in leaf #3 from B73 and W22 inbred lines. The red rectangles mark leaf tissue samples isolated for microscopy analysis. #3 leaves from W22 are slightly shorter than those form B73. We set the 0-cm position in W22 in the same way as in B73 and isolated samples in -4, 4, and 10 cm from the 0-cm position. **(H)** The distribution of gene expression correlations in B73, wild type control for *dct2* (*DCT2*), wild type control for *ppdk-1* (*PPDK*), *dct2*, and *ppdk-1* lines. Correlation coefficients for matching genes in the datasets were illustrated in the violin plots. In general, gene expression patterns over the four leaf developmental stages in B73 are conserved among the genotypes.


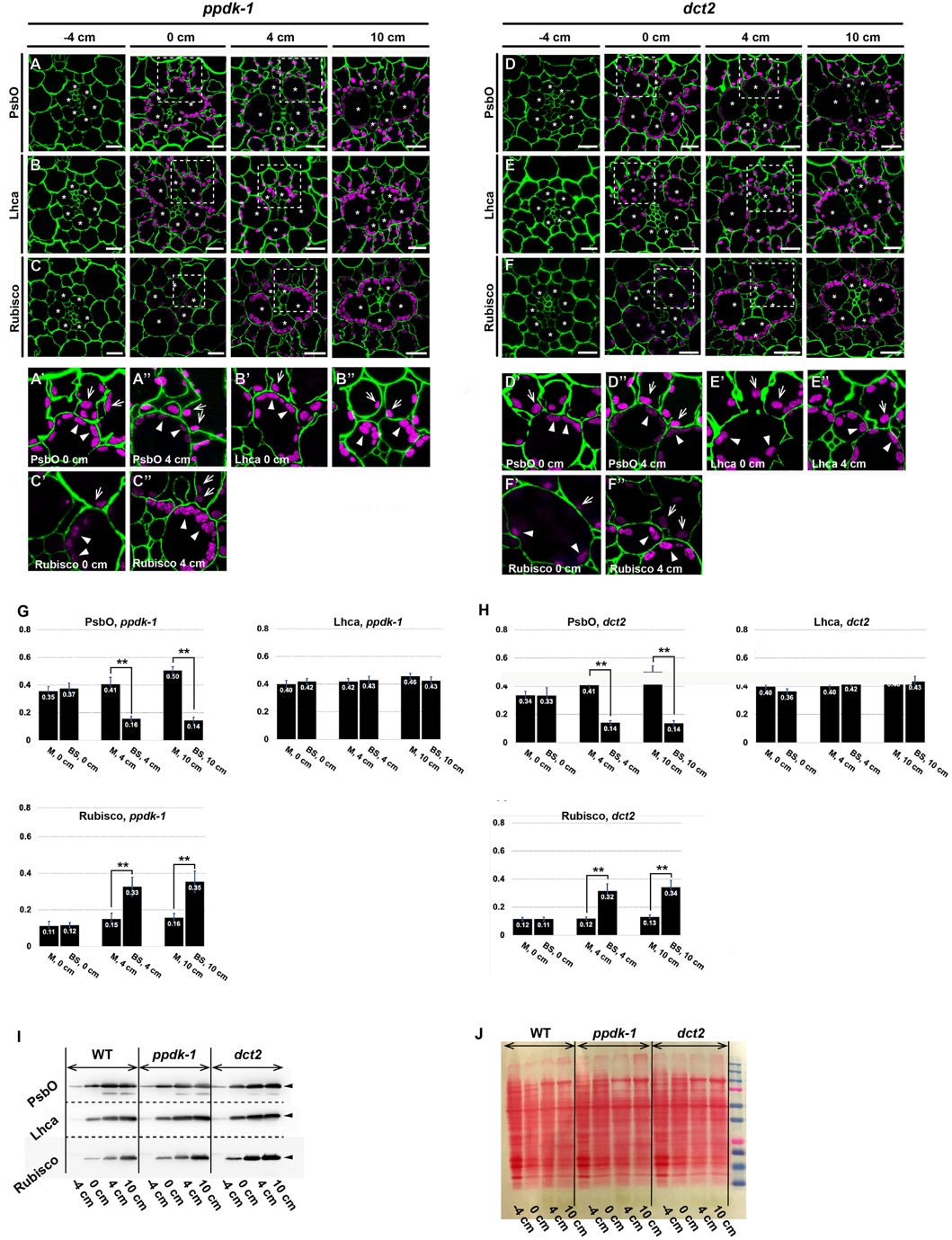


**Supplementary Figure S5.** Immunolocalization of chloroplast proteins in mesophyll (M) and bundle sheath (BS) cells of *ppdk-1* (A-C) and *dct2* (D-F) leaves.

**(A-F)** LR white sections from four leaf locations stained for PsbO (A and D), Lhca (B and E), and Rubisco large subunit (C-F). The cell wall was counter-stained to illustrate Kranz anatomy (pseudo-colored in green). Higher magnification images of boxed areas in 0 and 4 cm micrographs are presented below (A’-F’ and A”-F”). M and BS chloroplasts are marked with arrows and arrowheads in the magnified panels. Differential enrichment of PsbO and CURT1A in M chloroplasts was observed in 4-cm and 10-cm sections. Rubisco concentrated to BS chloroplasts in 4-cm and 10-cm sections. Lhca, a PSI subunit, accumulated evenly in the two types of chloroplasts. Scale bars: 10 µm.

**(G and H)** Average intensities were calculated from 20 randomly chosen M or BS chloroplasts in *ppdk-1* (G) and *dct2* (H) micrographs from three leaf samples. Error bars depict standard deviation (SD) (**, p<0.01 by Student t-test).

**(I and J)** Immunoblot analysis of the four proteins localized by immunofluorescence microscopy (I) and a nitrocellulose membrane stained with Ponceau S dye (J) showing that equal sample loading in the lanes. The blots in (I) were cropped but the original blots are presented in Supplementary Figure 8.


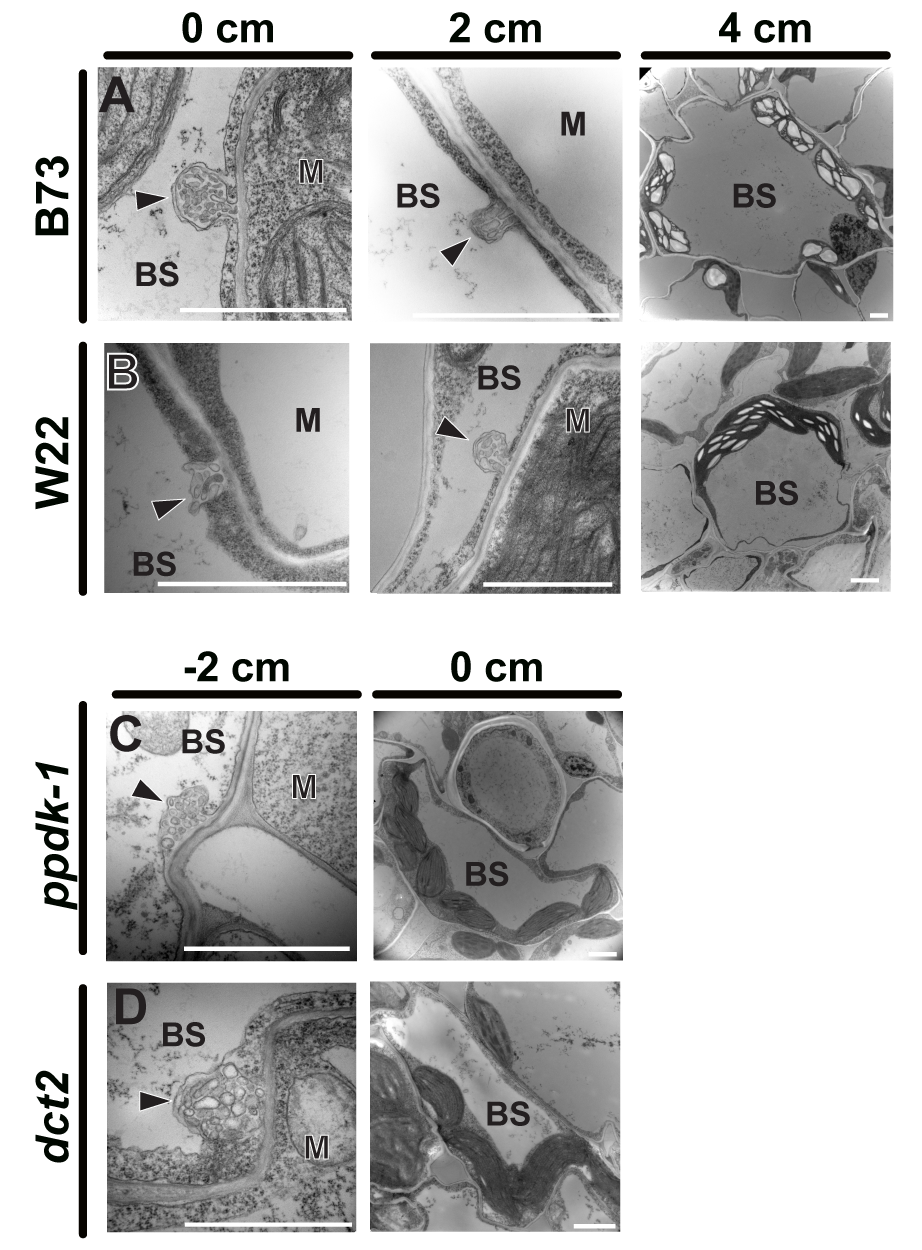


**Supplementary Figure S6**. TEM analysis of suberizing BS cells

TEM micrographs of BS and M cells in leaf samples from B73 **(A),** W22 **(B)** inbred lines and *ppdk-1* **(C),** *dct2* **(D)** mutant lines. Arrowheads indicate the plasma membrane ingrowths containing vesicular tubules in BS cells. In B73 and W22 leaves, the extracellular membrane structures were seen in 0 and 2 cm sections **(A and B)** while they were discerned in -2 cm leaf sections from the two mutant lines **(C and D).** The extracellular vesicular tubules disappeared in 4 cm and 0 cm sections from the inbred and mutant lines, respectively. Scale bars indicate 2 μm.

**
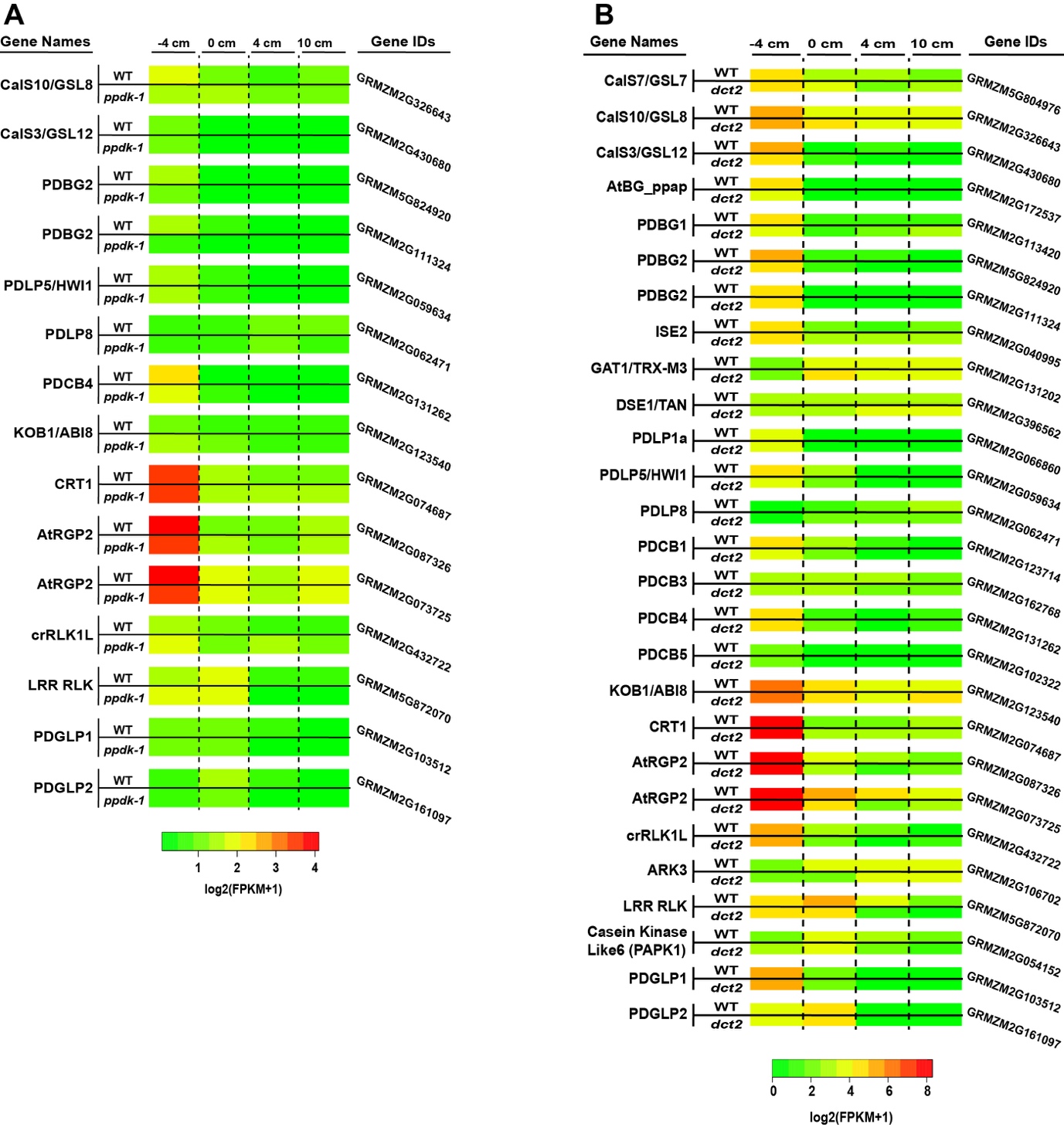
**

**Supplementary Figure S7.** RNA-seq analysis of genes for PD components

**(A and B)** Heat maps showing transcript levels of genes encoding PD components in *ppdk-1* (A) and *dct2* (B) in comparison with wild type (WT). PD genes with FPKM values higher than 1 are shown (SI Appendix, Dataset. S1). None of the PD genes displayed stronger transcriptional activities in *ppdk-1* or *dct2* than in WT.


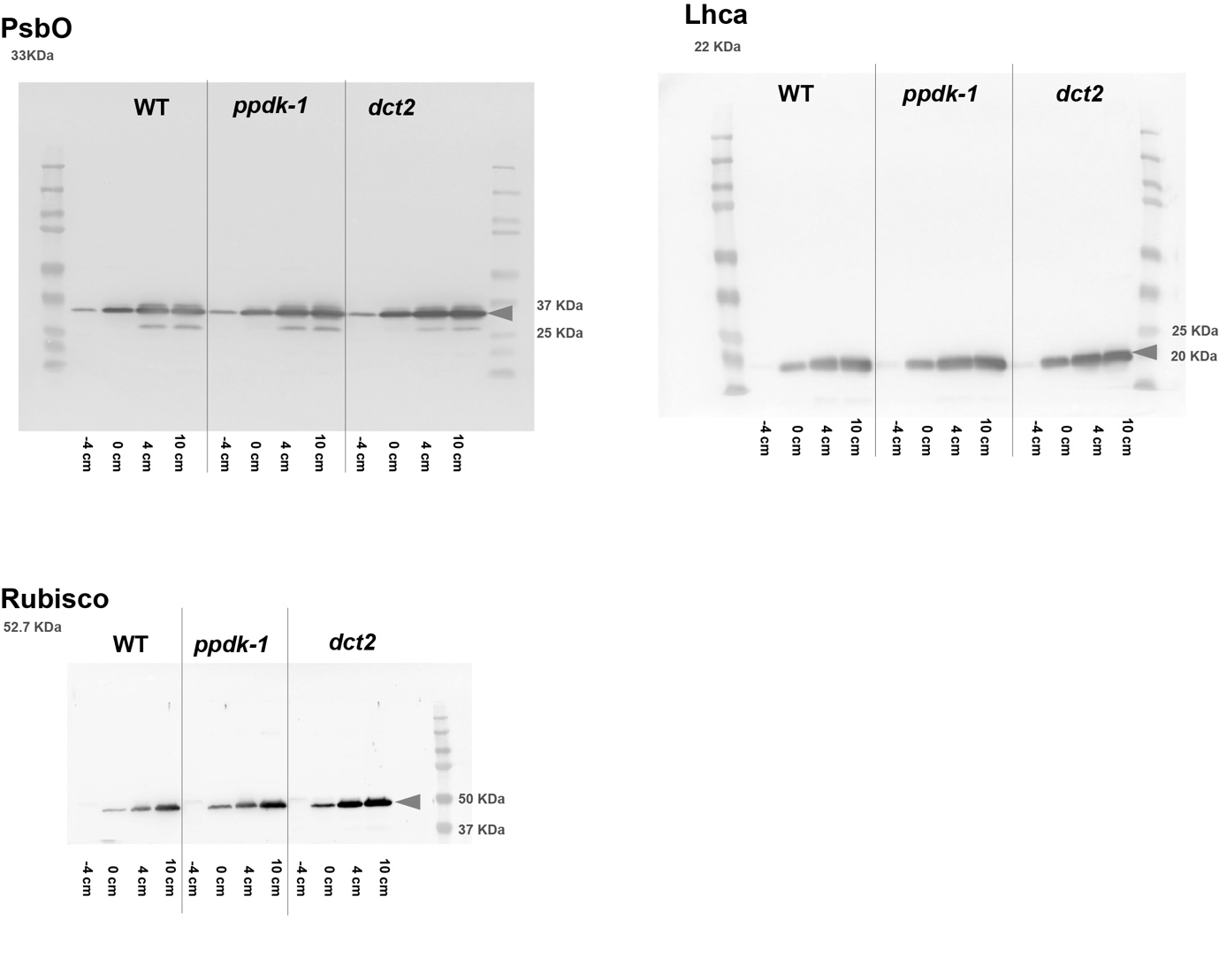


**Supplementary Figure S8.** Full immunoblots of the three chloroplast proteins in Figure S5I before cropping.

| Gene Name | Reference | Primer Sequences (5' to 3') | Size (bp) |
| --- | --- | --- | --- |
| CYP86B1/RALPH | GeneID: GRMZM2G162758 | Forward: CGAGTCCGCCTACAAGTTCA  Reverse: CTCGGCACCAACAGTTCAGA | 238 |
|  |  |  |  |
| ABCG | GeneID: GRMZM2G054332 | Forward: GACCTCACCAAGTGGGACTG  Reverse: CGAGCAGCAGCGAAATGTAG | 85 |
|  |  |  |  |
| GAPDH | GenBank: X07156.1 | Forward: TGGTTTCTACCGACTTCCTTGG  Reverse: TAGCCCCACTCGTTGTCGTA | 118 |

**Supplementary Table 1.** Primer sequences for qRT-PCR in Figure 6
